# Tandem-sgRNA Provides an Effective Reverse Genetic Approach for Suppression of *Streptomyces* Biosynthetic Gene Clusters and Secondary Metabolism

**DOI:** 10.1021/acssynbio.6c00274

**Published:** 2026-07-11

**Authors:** Chengxi Zhang, Nitya Kalyani Josyula, Ivette Cornejo-Corona, Rebecca Watson, José D. Cediel-Becerra, Marc G. Chevrette, Timothy P. Devarenne, Paul D. Straight

**Affiliations:** †Department of Biochemistry & Biophysics and ‡Graduate Program in Genetics and Genomics, 14736 Texas A&M University, AgriLife, College Station, Texas 77843, United States; § Department of Microbiology and Cell Science, 3463 University of Florida, Gainesville, Florida 32611, United States; ∥ Department of Plant Pathology and Wisconsin Institute for Discovery, University of Wisconsin-Madison, Madison, Wisconsin 53706, United States

**Keywords:** *Streptomyces*, secondary metabolism, CRISPR interference, biosynthetic gene cluster (BGC), antibiotics, carotenoids

## Abstract

Bacterial biosynthetic gene clusters (BGCs) encode secondary metabolites with diverse biological activities; however, most BGC products remain uncharacterized. One approach to identifying products and their metabolism is to use reverse genetics to identify metabolite-associated phenotypes. CRISPR interference (CRISPRi) offers a promising approach to disrupt BGC functions in high-GC genomes, typical of *Streptomyces* species. In many of these organisms, single-guide RNA (sgRNA)-mediated CRISPRi often results in incomplete product suppression, resulting in partial phenotypes that are unsuitable for functional studies. Using *Streptomyces* sp. Mg1, we found that a tandem-sgRNA configuration for CRISPRi improved the efficiency of target metabolite suppression. We engineered strains to express two sgRNAs to target the same promoter region within a BGC, resulting in greater than 80% metabolite suppression across diverse secondary metabolite classes. We used tandem-sgRNA CRISPRi to identify phenotypes associated with the loss of polyketide linearmycins, the siderophore desferrioxamine, the terpene β-carotene, and an uncharacterized nonribosomal peptide synthetase (NRPS). This approach revealed that β-carotene depletion substantially reduced intrinsic cellular autofluorescence. Targeting the unknown NRPS produced developmental phenotypes and enabled the identification of the biosynthetic genes for the antibiotic lavendomycin, revealing a noncollinear organization of genes in the BGC. We suggest that tandem-sgRNA CRISPRi provides an efficient reverse genetics platform for the functional characterization of *Streptomyces* BGCs, enabling the correlation of metabolites with the gene function, identification of associated phenotypes, and prioritization of cryptic BGCs for natural product discovery.

## Graphical abstract

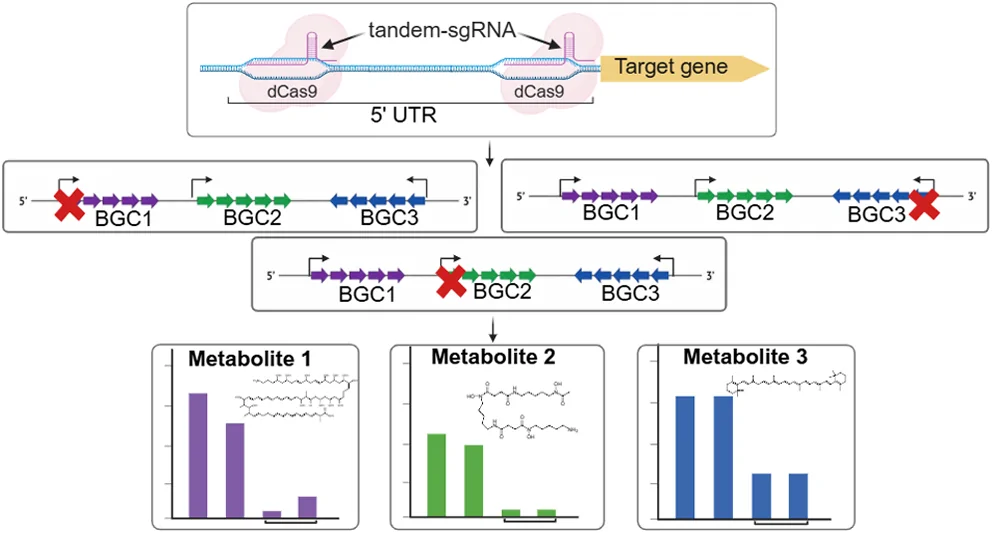

## Introduction

Bacterial secondary metabolites support environmental and interspecies interactions.^1–3^ Many of these small molecules are bioactive and have clinical or therapeutic benefits, including antibiotics, antivirals, anticancer drugs, and immunosuppressants.^4–6^ The enzymes for secondary metabolites are commonly encoded in large biosynthetic gene clusters (BGCs) within bacterial genomes.^7–9^ This gene organization enables powerful computational and experimental approaches for natural product discovery. Bioinformatic predictions have revealed that some bacteria encode an extensive secondary metabolome of 30 or more BGCs, most of which have yet to be explored.^10–13^ Therefore, a key goal for understanding the functions embedded in secondary metabolism is to identify new chemical structures and their associated biological activities from the genomes of diverse microbial species.^14,15^ These efforts benefit from experimental tools that facilitate the researcher’s ability to connect each BGC to its corresponding product metabolites and their activities, especially when the gene content fails to inform product prediction.

*Streptomyces* species are rich sources of specialized metabolites. Using bioinformatic tools, it is possible to estimate the secondary metabolome of an individual species by identifying its BGCs for known and unknown products. Two major challenges are to identify new metabolites and to determine the biological functions associated with the secondary metabolome of a given genome. One approach is to engineer genetic systems that force the expression of BGCs, either *in situ* or in heterologous hosts.^16–18^ A complementary but lesser-used approach is to genetically disrupt BGCs to identify loss-of-function phenotypes. In this reverse genetic approach, an efficient means to disrupt the targeted BGCs from *Streptomyces* would promote the discovery of new biological activities and phenotypes. However, genome manipulation of many *Streptomyces* species, particularly nonstandard model organisms, is challenging and inefficient, primarily due to their high-GC (commonly ≥ 70%) genomes, mycelial growth, low transformability, and intrinsic genetic instability.^19^

CRISPR-based genetic approaches have the potential to accelerate BGC-directed discovery of specialized metabolites. CRISPR-Cas9 from *Streptococcus pyogenes* and other CRISPR systems have been developed for *Streptomyces* genetics to delete, activate, or reversibly knockdown genes and BGCs.^20–24^ A selection of different configurations for CRISPR toolkits provides improved efficiency in strain engineering, but limitations persist. For example, CRISPR-Cas9 systems deliver targeted double-strand breaks (DSBs) in DNA, which are repaired by either homologous recombination or error-prone nonhomologous end joining (NHEJ).^20,25^ Both DSB repair pathways may lead to chromosomal deletions and rearrangements.^26,27^ Additionally, Cas9 expression can be toxic to bacteria, primarily due to off-target effects.^27,28^ An alternative to gene disruption is CRISPR interference (CRISPRi), which employs an enzymatically dead Cas9 (dCas9) enzyme as a roadblock to transcription.^29,30^ The CRISPRi approach has been successful in many studies on *Streptomyces* gene function.^22,23^ However, we found that CRISPRi in our model strain, *Streptomyces* sp. Mg1 (*S*. Mg1), resulted in minimal reductions of proteins and metabolites from the targeted genes and BGCs. A partial or minimal reduction in products is poorly suited for a reverse genetic approach, particularly when the product of the BGC is unknown.

Typical of *Streptomyces*, most *S.* Mg1 secondary metabolites have yet to be isolated or characterized. Analysis of the complete genome sequence using antiSMASH (antibiotics and secondary metabolite analysis shell)^31^ predicted 28 chromosomal BGCs and one plasmid-encoded BGC, including nonribosomal peptides (NRPs), polyketides (PKs), terpenes, and ribosomally encoded, post-translationally modified peptides (RiPPs) (Table S1). Two known *S.* Mg1 metabolites, chalcomycins/algdamycins^32^ and linearmycins,^33^ have been reported using chemical and genetic approaches, while a few others have been predicted based on conserved BGC sequences. Although the explicit metabolic products of most candidate BGCs are unknown, bioinformatic predictions indicate that many BGCs encode the synthesis of molecules with new structures to be identified.

We hypothesize that many of these yet uncharacterized secondary metabolites function either to support the growth and development of the producer population directly or to interfere with the growth of competitor species. Therefore, the goal of this study was to identify an efficient CRISPR-based approach to disrupt BGCs in *S.* Mg1 and reveal the phenotypes associated with the loss of the corresponding BGC products. Where relevant, strains with visible phenotypes may then be prioritized for the identification of the uncharacterized products and exploration of their functions. In this study, we focused on CRISPRi as an approach to generate loss-of-function phenotypes. We examined different configurations of single-guide RNAs (sgRNAs) to enhance CRISPRi metabolite depletion from the targeted *Streptomyces* BGCs. Our results demonstrated that targeting a single promoter with two tandemly directed sgRNAs for CRISPRi reliably yielded ≥80% reduction in product metabolite accumulation relative to controls. The improved repression from tandem-sgRNA CRISPRi provides loss-of-function phenotypes and detectable depletion of metabolites. We describe the application of tandem-sgRNA CRISPRi to multiple BGCs in *S.* Mg1 with known and unknown product metabolites. We selected four targets from the *S.* Mg1 genome that represent different biosynthetic classes. The targets include the polyketide linearmycins, the siderophore desferrioxamine, the terpene β-carotene, and a nonribosomal peptide synthetase (NRPS) with an uncharacterized product. Using this approach, we found that β-carotene depletion is associated with a substantial decrease of the intrinsic fluorescence in *S.* Mg1. The identification of a phenotype for the uncharacterized NRPS (BGC 28) led to the identification of a lavendomycin-like metabolite and its biosynthetic pathway.^34^ Our data suggests that tandem-sgRNA CRISPRi provides an efficient reverse genetics approach to reveal biological activities from BGCs in a *Streptomyces* genome, correlate metabolite masses with the loss of BGC function, and prioritize BGCs for further genetic and biochemical studies.

## Results

### Integrating CRISPRi Vector for the Disruption of *Streptomyces* Genes

Our initial attempts to use CRISPR in *S.* Mg1 resulted in variable and low efficiency of gene deletions (Table S2); therefore, we sought to use CRISPRi as a primary screening tool for BGC disruption phenotypes. To improve the stability of CRISPRi over temperature-sensitive replicating vectors, we first engineered a vector to stably integrate CRISPR genes into the chromosome. Using components of the pCRISPomyces-2 plasmid,^20^ we constructed a pSET152-based CRISPRi plasmid (pCZ2) that expressed a catalytically inactive dCas9 with point mutations D10A and H840A from the constitutive promoter P*rpsL* (XC). The construct, pCZ2, expresses a single-guide RNA (sgRNA) cassette from a constitutive P*gapdh* (EL) promoter (Figure 1). The pCZ2 plasmid also carries an integrase and the ϕ-C31*attP* sequence for insertion into the host *attB* locus.^35^ To test the function of pCZ2, we designed sgRNAs to target two BGCs in *Streptomyces coelicolor*, actinorhodin (ACT), and undecylprodigiosin (RED), for which previous reports demonstrated efficient CRISPRi.^36^ *S. coelicolor* carrying pCZ2 with 20-nucleotide sgRNAs targeting either the *redD* (activator of RED cluster) promoter, the gene *actI-ORF1* (biosynthetic gene of ACT cluster), or the gene *actII*-4 (activator of ACT cluster), visibly reduced the production of their corresponding pigmented products (Figure 2). The process to generate, cure, and verify the engineered strains took 2 weeks, which is favorable for use as a genome-wide approach. Having demonstrated the use of pCZ2 for CRISPRi in *S. coelicolor*, we next sought to determine its functionality using multiple targets in the *S.* Mg1 genome.

**Figure 1:**
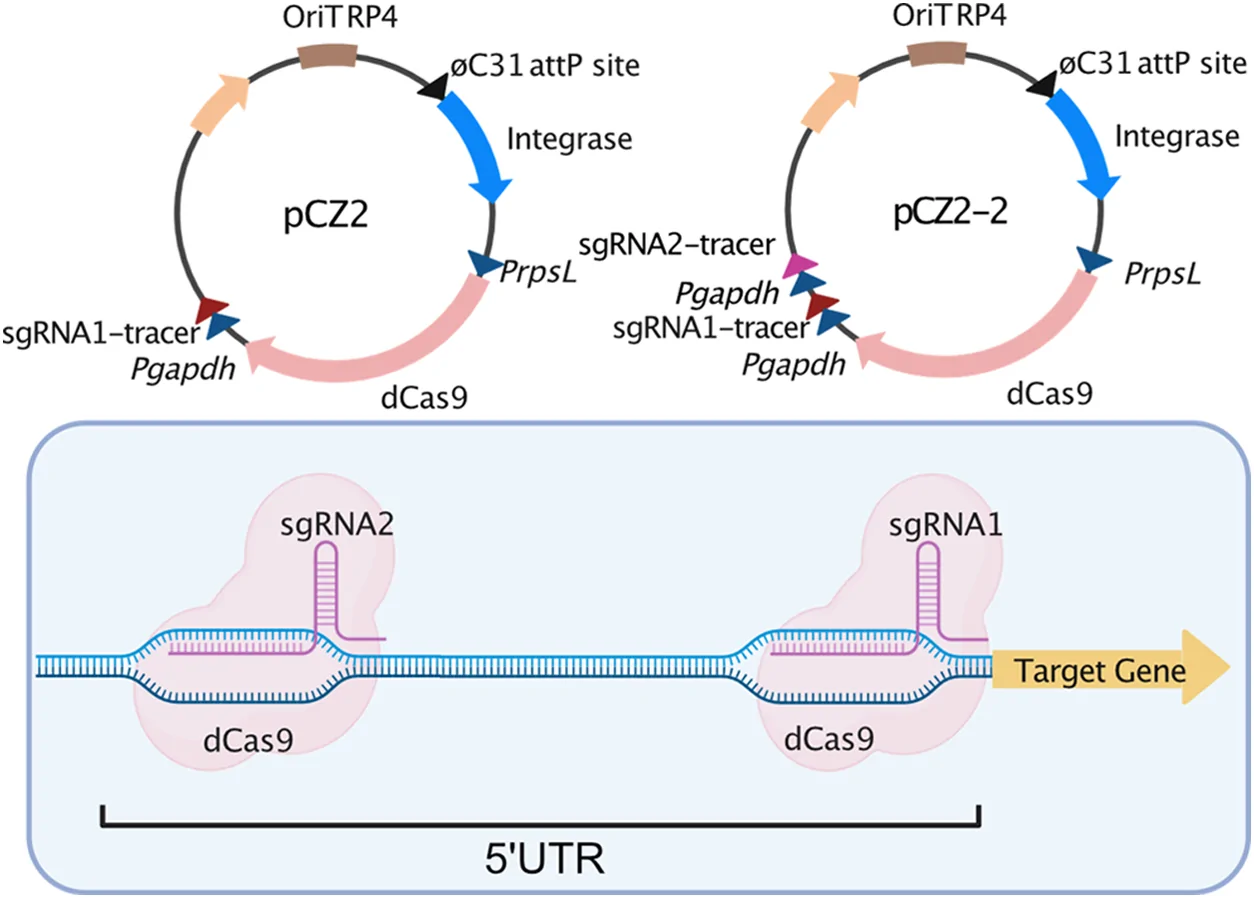
Diagrams of pCZ2 CRISPRi plasmids. Diagrams of the pCZ2 CRISPRi plasmids that constitutively express dCas9 and one (pCZ2) or two tandem (pCZ2-2) guide RNAs, each controlled by the P*gapdh* promoter. A sketch of the 5′UTR of a targeted gene using pCZ2-2 to illustrate the tandem arrangement of guide RNAs and dCas9 binding sites.

**Figure 2:**
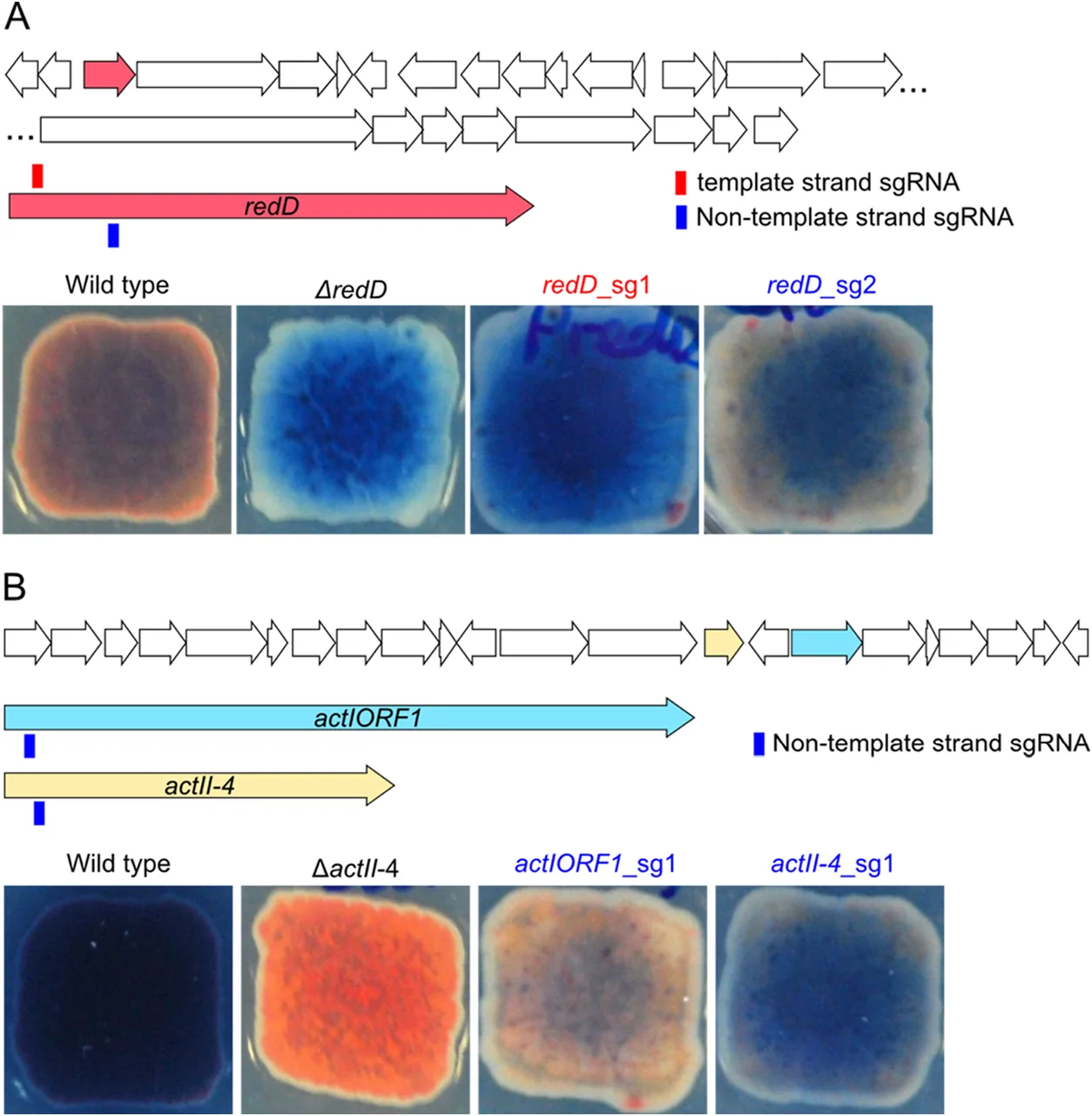
CRISPRi disruption of *S. coelicolor-*pigmented antibiotics. (A) CRISPRi using pCZ2 to target the RED BGC. The red bar indicates a template-stranded sgRNA, while the blue bar indicates nontemplate-stranded sgRNAs. Cell patches on agar media reveal phenotypes. The template-stranded sgRNA *redD*_sg1, targeting near the *redD* promoter, abolished RED production. The nontemplate-stranded sgRNA *redD*_sg2, targeting within *redD* ORF, partially reduced RED. The photo was taken after 48 h of incubation on R2YE agar. A Δ*redD* strain served as a control. (B) CRISPRi using pCZ2 to target the ACT BGC. The *actI*-ORF1 sgRNAs targeted the biosynthetic gene *actI*-ORF1, and the actII-4_sg1 sgRNA targeted the pathway-specific regulator *actII-*ORF4.^22^ A Δ*actII-*ORF4 strain served as a control. The *actI*-ORF1 sgRNA strain showed dramatically reduced ACT production, while the *actII-*ORF4 sgRNA showed a reduced effect. The photo was taken after 72 h of incubation on R2YE agar.

To determine the efficacy of pCZ2 in *S*. Mg1, we initially targeted the surfactin hydrolase gene, *sfhA*, for which a phenotypic assay is available to identify mutants.^37^ On agar plates where both species are present, surfactin hydrolase protects the development of *S.* Mg1 aerial hyphae from the disruptive effects of *B. subtilis* surfactin (Figure 3). In the absence of *sfhA*, *S.* Mg1 cannot produce aerial hyphae due to the inhibition by surfactin from *B. subtilis*. The complete deletion strain (Δ*sfhA*) showed a circular region without aerial hyphae, measuring 9.8 ± 0.05 mm in diameter. Using a pCZ2-generated *sfhA-*targeting CRISPRi strain, we found that the affected region of *S.* Mg1 diminished to 3.7 ± 0.05 mm in diameter (Figure 3). The results indicated that the CRISPRi reduced surfactin hydrolase production, but the intermediate phenotype relative to the controls suggested only partial repression of *sfhA* expression.

**Figure 3:**
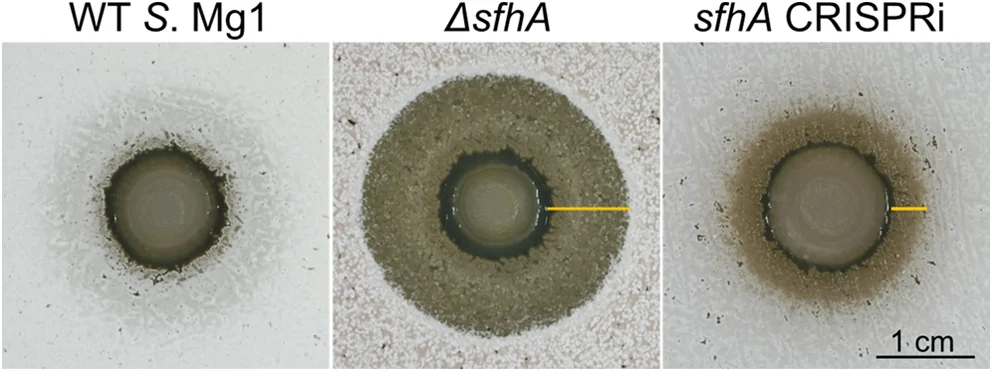
CRISPRi partially reduced surfactin hydrolase production in *S.* Mg1. When cocultured on MYM agar, surfactin produced by wild-type *B. subtilis* (center) is hydrolyzed by surfactin hydrolase produced by *S*. Mg1 (periphery) spread on the agar surface. The presence of the SfhA enzyme enables wild-type *S*. Mg1 to develop aerial hyphae (white surface) (WT *S.* Mg1). Deletion of *sfhA* leads to the blockage of *S.* Mg1 development by surfactin, resulting in a bald zone with a 9.8 ± 0.05 mm diameter (Δ*sfhA*). Targeting surfactin hydrolase with CRISPRi resulted in an intermediate phenotype, with a bald region of 3.7 ± 0.05 mm in diameter (*sfhA* CRISPRi). The yellow lines mark the length of the measured bald regions. Bar is 1 cm.

## Tandem-sgRNA CRISPRi knockdown of linearmycins production in *S*. Mg1

We selected a second target for CRISPRi, the BGC encoding the biosynthesis of the linearmycin secondary metabolites by *S.* Mg1. Linearmycin synthesis is encoded by a ∼180-kilobase polyketide synthase (PKS) BGC^38,39^ (Figure 4A) (Table S1). The large multimodular biosynthetic ORFs are transcribed downstream of the *lnyHA* promoter in one giant operon (151 kilobases). The oppositely oriented *lnyI* gene encodes an acyltransferase that loads the first substrate in the linearmycin biosynthetic pathway. Our initial step was to select sgRNAs that targeted the functions necessary for linearmycin biosynthesis. Ideally, a single transcriptional unit should be targeted for a complete CRISPRi-mediated shutdown of BGC expression and product biosynthesis. The *lnyI* gene is essential for linearmycin production, thus prioritizing it for CRISPRi.^38^ We also selected the *lnyHA* promoter as the transcriptional regulator for the primary operon. We designed three sgRNAs to target 1) *lnyI*, 2) the *lnyHA-*predicted promoter region (*lnyHA*-sg1), and 3) near the *lnyHA* translation start site (*lnyHA-*sg2) (Figure 4B).

**Figure 4:**
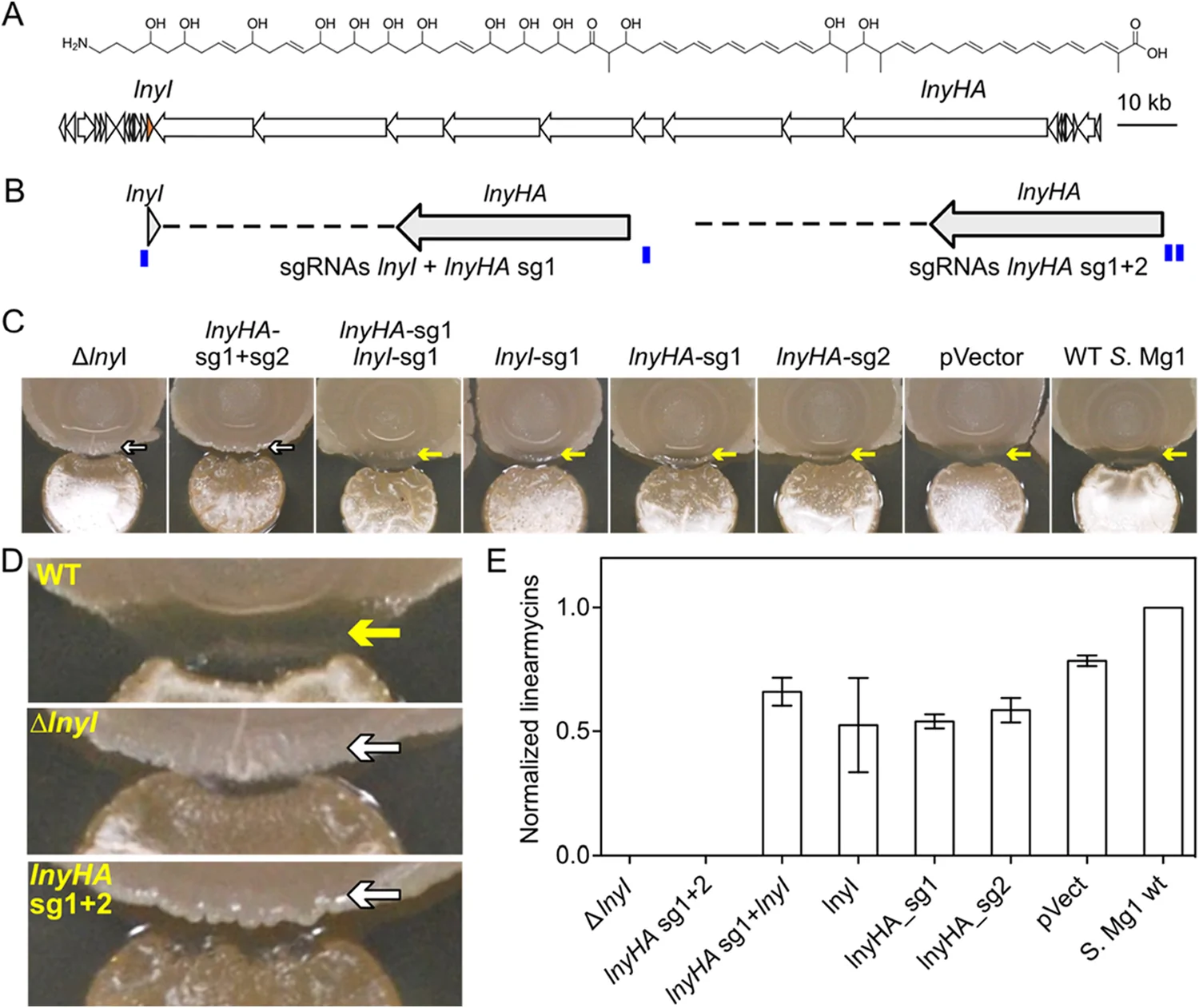
CRISPRi disruption of linearmycin production by *S.* Mg1. (A) Structure of linearmycin B and a sketch of the ∼180kb linearmycin BGC. (B) Relative positions of sgRNAs directed toward linearmycin genes. The blue lines indicate the nontemplate-strand sgRNAs. One strain expressed dual-sgRNAs to target the promoters of *lnyI* and *lnyHA* (left). One strain expressed tandem sgRNAs to target the promoter of *lnyHA* (right). (C) Biological assay of linearmycin activity using *B. subtilis* (upper) and *S.* Mg1 (lower) colonies. The edge of the *B. subtilis* colony proximal to *S.* Mg1 is lysed by linearmycins, resulting in a clear zone (yellow arrows). Mutations that disrupt linearmycin production prevent lysis and clearing zones (white arrows). (D) Magnified images from (C) for WT, Δ*lnyI*, and lnyHA-sg1 + 2 to enhance the visibility of the lysed *B. subtilis* region. (E) Quantitation of linearmycins from HPLC analysis of culture extracts. Area under the curve for each sample relative to the wild-type extracts. Three replicate extracts were used for each strain. Peaks were detected using UV at 333 nm.

We compared and quantified the production of linearmycins between the control strains and different configurations of the CRISPRi strains using a *B. subtilis* cell lysis assay and high-performance liquid chromatography (HPLC). Using the cell lysis assay, only a minor reduction in *B. subtilis* lysis was observed compared to wild-type *S.* Mg1 for strains with sgRNAs *lnyI*-sg1, *lnyHA*-sg1, or *lnyHA*-sg2, indicating minimal suppression of the linearmycins by CRISPRi (Figure 4C). Accordingly, we detected only a modest reduction in linearmycins by HPLC in all three CRISPRi-targeted strains compared to the controls (Figure 4E). Like *sfhA*, sgRNA-directed CRISPRi of linearmycins results in partial knockdown phenotypes with moderately reduced product formation. Using qRT-PCR, we found no significant reduction in *lnyHI* or *lnyHA* transcript abundance with a single-guide RNA (Table S5). We concluded that a single sgRNA-directed CRISPRi is insufficient as a roadblock to transcription for providing reliable depletion phenotypes. Thus, we sought an approach to improve the efficacy of CRISPRi.

Multiplex-sgRNA CRISPRi was used to target multiple BGCs in a *Streptomyces* genome.^23^ We hypothesized that adding a second guide RNA within a BGC may have an additive effect on reducing product formation. A dual-sgRNA approach in eukaryotic cells has been reported to improve the CRISPRi of target genes.^40^ We engineered a plasmid, pCZ2-2, to simultaneously express two sgRNAs, each controlled by the gapdh (EL) promoter element (Figure 1). We engineered the plasmid to express either dual-sgRNA (two different transcriptional units) or tandem-sgRNA (the same transcriptional unit) configurations (Figure 4B). The dual sgRNAs targeted two independent promoters, *lnyI* and *lnyHA*. The tandem sgRNAs both targeted the upstream region of *lnyHA* (*lnyHA-*sg1 and *lnyHA-*sg2), with sg1 targeting the predicted promoter sequence and sg2 targeting nearer to the translation start site (RBS) (Figure S6). Using the cell lysis assay against *B. subtilis*, we found that the dual-sgRNA strains (*lnyHA-*sg1 and *lnyI*) targeting different promoters retained lytic activity and that linearmycins were still produced (Figure 4C). However, using the tandem *lnyHA*-targeting strain (*lnyHA*-sg1 and *lnyHA*-sg2), we observed a nearly complete disruption of linearmycin production by both bioassay (lysis of *B. subtilis*) and HPLC (Figure 4C–E). The results revealed that two sgRNAs operating in tandem to target a single promoter improved the repression of BGC expression and prevented the subsequent formation of the corresponding product metabolite. Using quantitative RT-PCR to assess the impact of tandem sgRNAs on targeted transcript abundance, we observed significant transcript repression for both *lnyHA* and *lnyHI* transcripts relative to the vector and single-sgRNA controls (Table S5). Thus, we conclude that the primary impact of tandem-sgRNA is to improve the suppression of target gene transcription. To determine whether tandem-sgRNA CRISPRi functions effectively for multiple BGCs, we selected three additional targets based on their different biosynthetic mechanisms: desferrioxamines, isorenieratene, and an NRPS with an uncharacterized product (Table S1, clusters 11, 2, and 28).

### CRISPRi Knockdown of Desferrioxamine Production in *S*. Mg1

We next targeted the desferrioxamine BGC (Table S2, Cluster 11). Desferrioxamines are specialized metabolites that function as iron-scavenging siderophores produced by many *Streptomyces* species.^41^ We designed single and tandem sgRNAs (*desA*-sg1 and *desA*-sg2) targeting the upstream region of the *desA* gene in the desferrioxamine BGC and monitored product accumulation by HPLC (Figure 5 and S6). In a pattern similar to that observed for the linearmycins, the tandem-sgRNA CRISPRi strain substantially reduced desferrioxamines (≥90%), but the single-sgRNA CRISPRi controls had a relatively minor influence (Figure 5 and S1). An observable phenotype for the loss of desferrioxamines is the use of chrome azurol S in agar media.^42,43^ Using this assay, we observed the CRISPRi depletion phenotype for desferrioxamines (Figure S1). Collectively, the phenotypic and quantitative data suggest that the tandem-sgRNA CRISPRi may be useful for the knockdown of many targeted BGCs within a single genome. In both linearmycins and desferrioxamines, one of the sgRNAs, *desA*-sg1, targeted dCas9 near the −10 box in the promoter. The second sgRNA, *desA*-sg2, was located near the translation start site for the proximal gene or BGCs. To examine the effects of CRISPRi on other BGC targets, we used a similar configuration of tandem sgRNAs.

**Figure 5:**
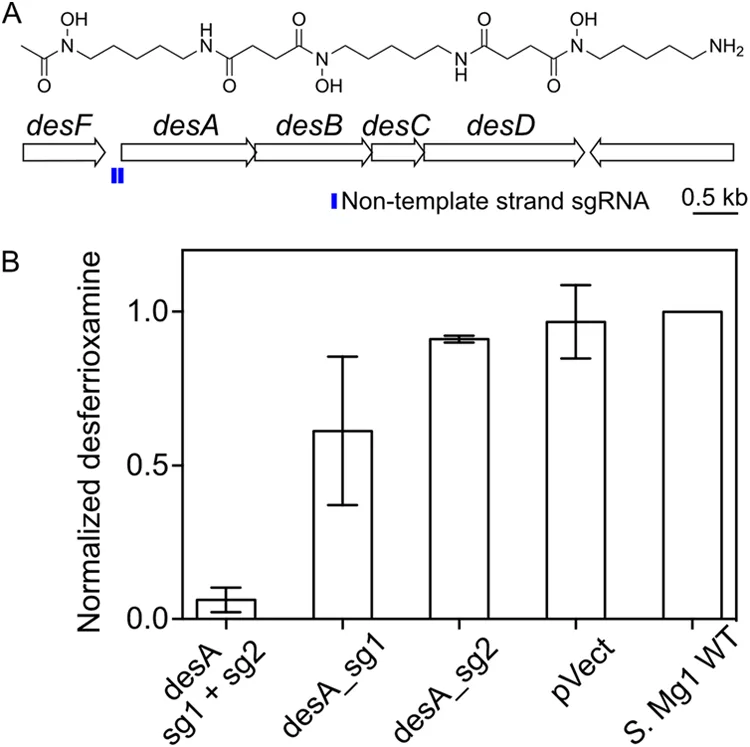
Tandem-sgRNA CRISPRi disrupts desferroxamine production in *S.* Mg1. (A) Structure of desferrioxamine B and a sketch of the BGC from *S.* Mg1. The blue lines indicate the relative position of tandem sgRNAs to target the promoter of *desA*, the first gene in the *desA-D* operon. (B) Quantification of desferrioxamine production by HPLC. Each column represents the average amount of desferrioxamine in triplicate samples, relative to that in wild-type *S.* Mg1. The detection wavelength was UV 430 nm.

### Tandem-sgRNA CRISPRi Depletion of β-Carotene Reduces the Background Fluorescence in *S.* Mg1 Cells

Many species of bacteria produce carotenoids like β-carotene and isorenieratene, although the physiological functions of these pigments are unclear. *S.* Mg1 encodes a carotenoid BGC (*crt*) (Table S1, cluster 2) similar to those in green sulfur bacteria^44^ and other *Streptomyces* species.^45–48^ The cluster, like the *S. griseus crt* BGC,^45^ encodes 7 biosynthetic enzymes in two oppositely directed transcriptional units (Figure 6A). The antiSMASH product predicted is isorenieratene (Figure 6B). We targeted the *crt* BGC for tandem-sgRNA CRISPRi. We selected the promoter region (142797–143032) that controls the transcription of three genes: *crtY*—lycopene cyclase, *crtT*—methyltransferase, and *crtU*—isorenieratene synthase, which are essential enzymes for the conversion of lycopene to β-carotene and isorenieratene.^49^ We designed two sgRNAs, *crtY*-sg1 and *crtY*-sg2, for CRISPRi, targeting the promoter region of the lycopene cyclase gene, *crtY*, and engineered strains that possessed either single or tandem sgRNAs targeting the promoter (Figure S6).

**Figure 6:**
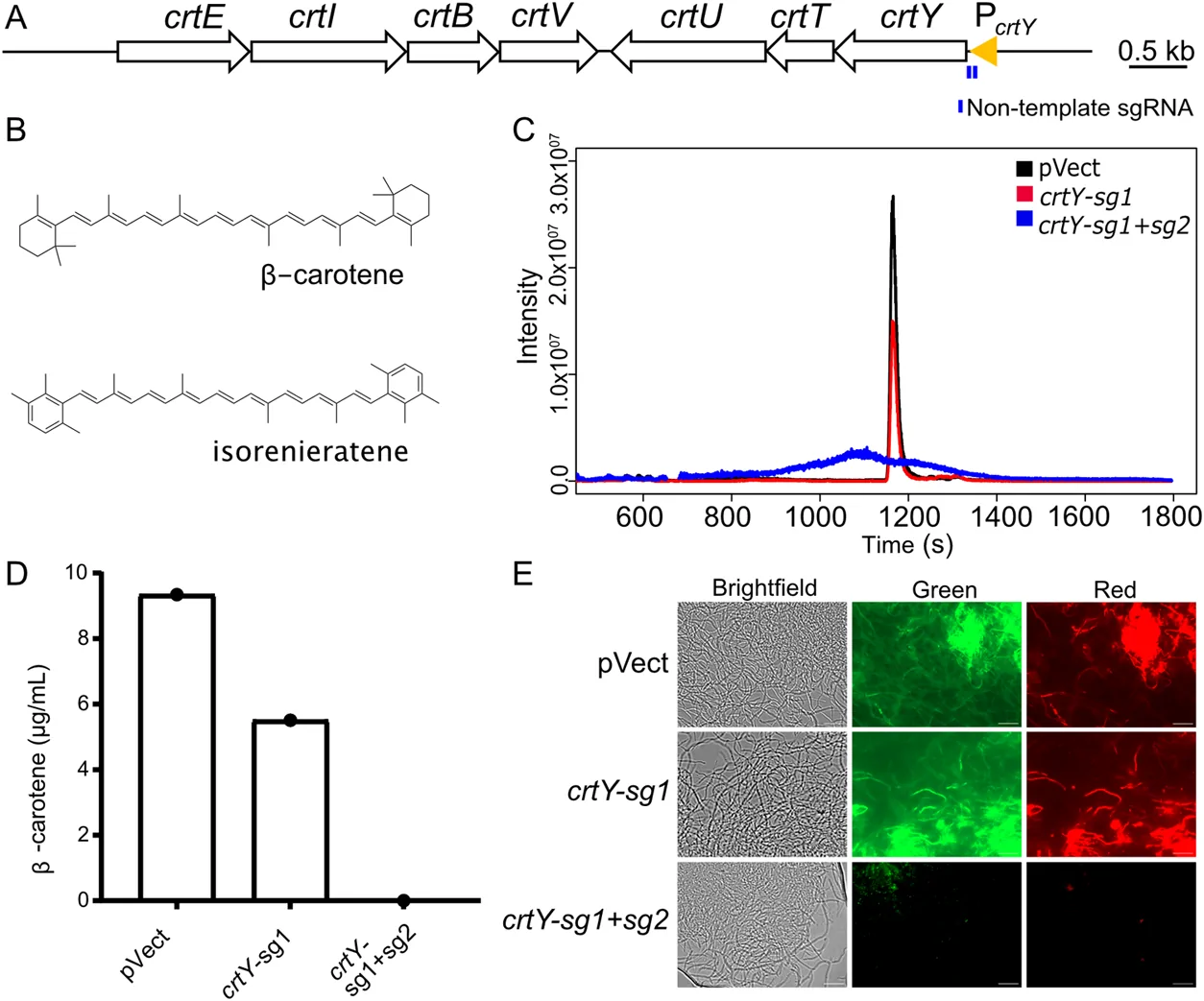
Tandem-sgRNA CRISPRi disrupts carotenoid biosynthesis to reveal background fluorescence signals. (A) Schematic representation of the carotenoid BGC from *S.* Mg1. The blue lines indicate the relative position of the tandem sgRNAs targeting the promoter of *crtY*. (B) Structural diagrams of β-carotene and isorenieratene, the predicted products of the carotenoid BGC. (C) LC/MS intensity of β-carotene (536.44) extracted from *S.* Mg1 strains: pVect, vector control; *crtY-sg1*, single-sgRNA CRISPRi; *crtY-sg1+sg2*, tandem-sgRNA CRISPRi. (D) Quantitative comparison of β-carotene production by *S.* Mg1 strains. The β-carotene content in the extracts was determined by comparing it to a standard curve of pure β-carotene by LC-MS. (E) Comparative fluorescence from imaging of *S.* Mg1 mycelia (brightfield) and autofluorescence (green—Ex: 469 nm; Em: 525 nm; red— Ex:531; Em: 647 nm) in control and CRISPRi strains.

We compared the effects of tandem-sgRNA versus individual sgRNA CRISPRi on growth defects and carotenoid production. The targeted CRISPRi strains showed a mild delay in colony development and sporulation relative to both vector and single-sgRNA controls, which we speculated may have been due to the loss of β-carotene. We analyzed crude extracts from wild-type cultures using LC-MS/MS and detected β-carotene ([M]^+•^ 536.44) as the predominant product of the *crt* cluster under the culture conditions used (Figure S2). We confirmed its identity using LC-MS/MS analysis of the parent ion and by comparing it with fragments of a pure β-carotene sample. We used pure β-carotene to generate a standard curve and quantified β-carotene from wild-type and CRISPRi samples (Figures 6C, D and S2), revealing a complete disruption of β-carotene in strains containing the tandem-sgRNA CRISPRi. Despite the loss of β-carotene production, the LC-MS chromatogram of the tandem-sgRNA CRISPRi strain showed an elevated baseline in the extracted ion chromatogram (Figure 6C). We suspected that targeting *crtYTU* genes for CRISPRi may lead to the accumulation of the β-carotene precursor, lycopene, which has the same mass as β-carotene (536.89 g/mol). Tandem LC-MS/MS of fragments from the residual mass confirmed its identity as a lycopene (Figure S2). Thus, our data indicate that tandem-sgRNA CRISPRi effectively blocks the conversion of lycopene to β-carotene in *S.* Mg1.

Although the phenotype was mild, we observed a slight delay in growth with the tandem-sgRNA of *crtYTU* compared to the vector-only control strain. This phenotype suggested that the loss of β-carotene may directly impact growth, as opposed to an issue related to dCas9 expression or off-target effects. β-Carotene is weakly fluorescent (green-yellow, 500–580 nm), and lycopene exhibits orange-red fluorescence (570–610 nm).^50,51^ We hypothesized that the depletion of β-carotene by tandem-sgRNA CRISPRi may be visible from changes in the cellular fluorescence of carotenoids. We imaged wild-type and tandem-sgRNA CRISPRi *crtYTU* cells at a higher resolution using light and fluorescence microscopy to detect any changes in the cell morphology or fluorescence following β-carotene depletion. The CRISPRi *crt* depletion strain showed no discernible differences in the cell size or morphology (Figure 6E). However, we observed changes in the fluorescence in both green (Ex 469/35 nm, Em 525/39 nm) and red (Ex 531/40 nm Em 647/57 nm) when comparing tandem-sgRNA CRISPRi mycelia to the control mycelia (Figure 6E). Although the single-sgRNA CRISPRi strain (*crtY-*sg1) had moderately reduced red fluorescence relative to the control, the strain containing tandem sgRNAs (*crtY*-sg1 + sg2) imaged under identical conditions had a minimal fluorescence signal. Because carotenoids have weak green fluorescence, the results suggest that the reduction in intrinsic green and red fluorescence is a secondary consequence of β-carotene depletion. The identity of the fluorescent compounds is yet to be determined, but other possible sources include redox cofactors.^52,53^ Our data support the conclusion that tandem-sgRNA CRISPRi effectively blocks β-carotene production by *S.* Mg1, producing an observable phenotype from a mild growth delay and a substantial reduction in intrinsic cellular fluorescence.

### Identification of a Lavendomycin-Like BGC Using Tandem-sgRNA CRISPRi

Our next goal was to target a BGC with an uncharacterized metabolite as the product. We selected an NRPS (Table S1, BGC 28) and engineered tandem-sgRNA CRISPRi targeting constructs using the predicted promoter region for the first ORF in the BGC (*ORF-A*) (Figure 7 and S6). We screened the CRISPRi-targeted strains for visible differences in growth, colony morphology, pigmentation, and sporulation by comparing the wild-type and vector control strains when cultured on agar media. After 2–4 days of incubation, we observed a moderate deficiency in growth and spore development using the tandem-sgRNA CRISPRi strain, *ORF-A* sg1 + sg2, while the controls and other sgRNA CRISPRi configuration strains showed minimal or no visible phenotypes (Figure 7). On the basis of the phenotypic results for the *ORF-A* sg1 + sg2 tandem-sgRNA CRISPRi strain, we hypothesized that the peptide product may function in coordinating growth, development of aerial hyphae, and spore formation.

**Figure 7:**
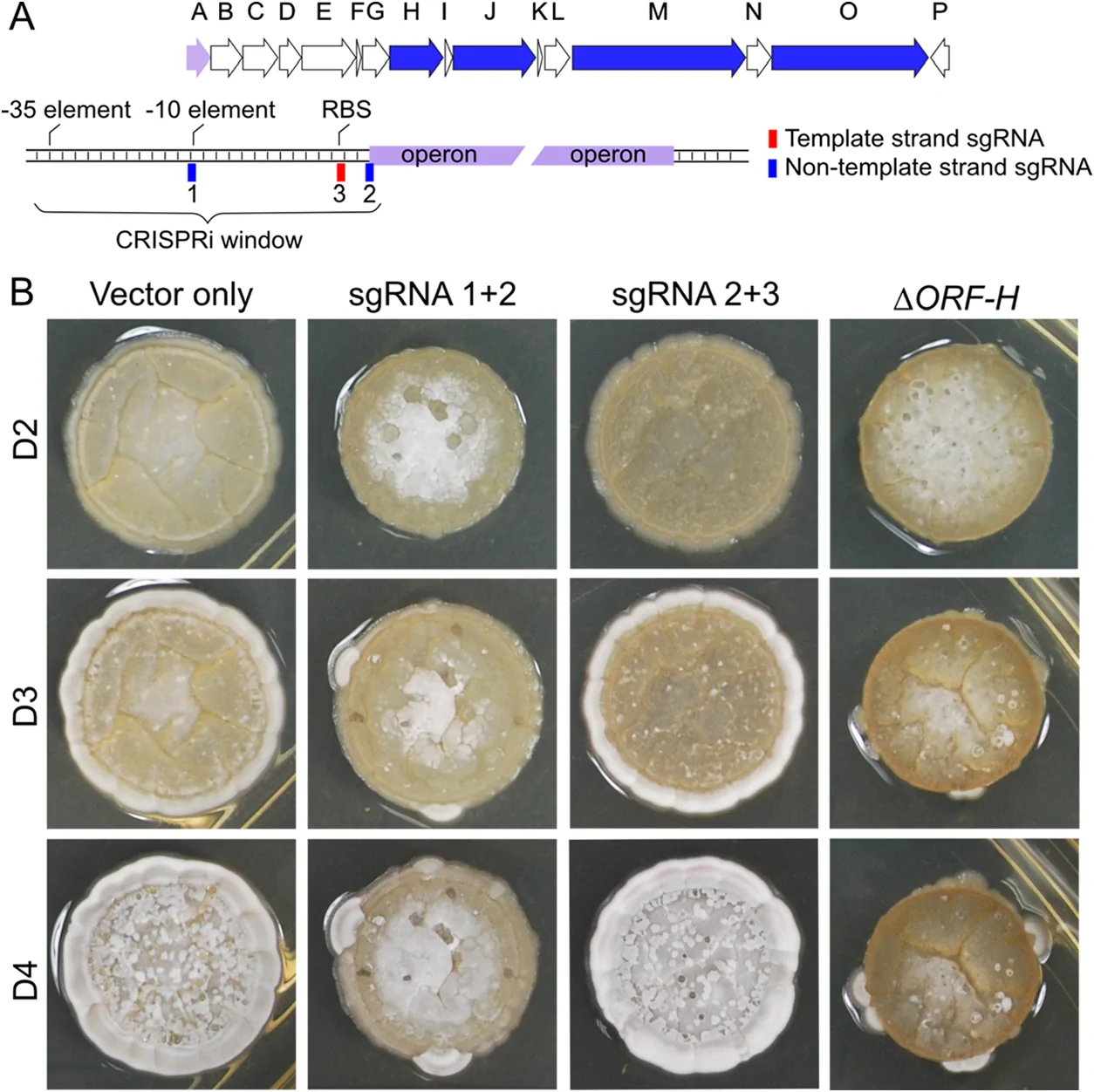
Tandem-sgRNA CRISPRi of NRPS reveals colony developmental defects in *S.* Mg1. (A) Schematic representation of the cluster 28 BGC from *S.* Mg1 and the upstream region of *ORF-A*, where tandem-sgRNA targets gene expression. The blue lines indicate the relative positions of nontemplate-strand sgRNAs. The red lines indicate the relative position of the template-strand sgRNA. (B) Comparison of colony morphology on day 2 (D2), day 3­(D3), and day 4­(D4), during which the development of aerial hyphae is observed as a white surface on the colony. The sgRNA 1 + 2 tandem-sgRNA strain shows aberrant development of aerial hyphae and colony outgrowth. The vector control and sgRNA 2 + 3 strains show normal development. A frameshift deletion of the *ORF-H* gene results in a colony defect similar to that of the sgRNA 1 + 2 tandem-sgRNA CRISPRi strain.

Since we observed a phenotype with tandem-sgRNA CRISPRi, we next sought to identify the corresponding metabolite product. Using pCRISPomyces-2, we engineered a deletion in *ORF-H*, which is predicted to encode the initiating adenylation domain required for the NRPS. The *ORF-H* deletion generates a frameshift in the downstream coding sequence and should abolish product formation, providing a control for the loss of NRPS function. The deletion of *ORF-H* produced a morphological phenotype similar to that of the *ORF-A* tandem-sgRNA CRISPRi strain (Figure 7). To identify the product metabolite, we performed comparative mass spectrometry on extracts from wild-type and BGC-disrupted strains. Using LC-MS and MALDI-TOF mass spectrometry, we identified an ion, *m*/*z* 667.4 (M+H)^+^, that was minimal or absent in the disruption strain (Figure S3), absent in the *ORF-H* deletion strain, and present in the control strains (Figure 8). We generated tandem MS spectra for the candidate ions. The primary mass and fragment ions from the NRPS BGC product matched those of the peptide antibiotic lavendomycin (666.78 g/mol)^34^ (Figures 8 and S4). Therefore, we provisionally designated the genes in the BGC *lvdA-P* based on their proposed functions in the synthesis of a metabolite related to lavendomycin. We compared the tandem-sgRNA CRISPRi strains with control strains to measure relative metabolite production using LC-MS. We observed that the *ORF-A­(lvdA)* sg1 + sg2 configuration of sgRNAs targeting the promoter diminished the product metabolite by 80–90% (Figure 8). As previously done for linearmycins, we assessed the impact of tandem sgRNAs on *lvdA* transcript abundance to determine whether improved repression of transcription was the likely cause of the observed phenotypes. We observed a several-fold reduction in transcript abundance in the tandem-sgRNA strain, while the control strain exhibited a minor elevation in the transcript abundance relative to the vector control (Table S6). This level of product suppression from tandem sgRNAs resulted in a phenotype similar to that of the deletion mutant, despite some residual peptide production in the CRISPRi strain.

**Figure 8:**
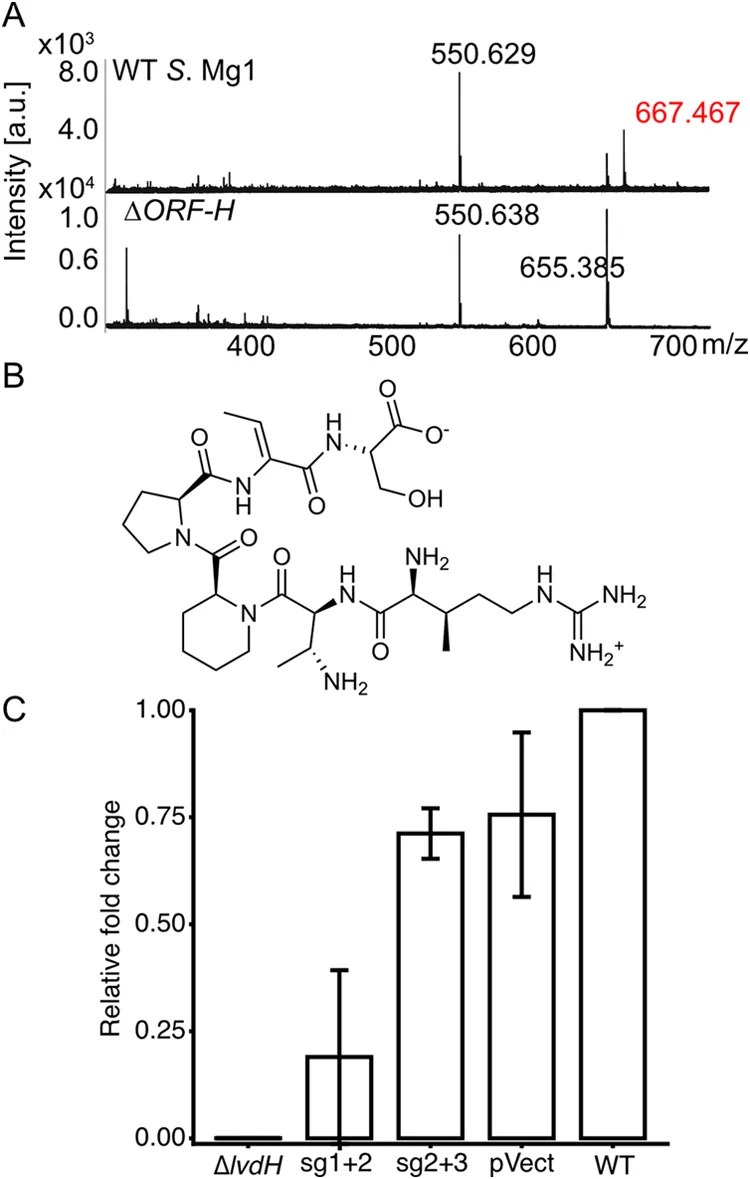
Diminished production of lavendomycin-like metabolites by tandem-sgRNA CRISPRi. (A) MALDI-TOF MS spectra of WT and Δ*ORF-H S.* Mg1. The peak at 667.467 [M + H]^+^ is absent in the extracts of the mutant strain. (B) Structure of lavendomycin. (C) Intensity of the lavendomycin-like metabolite (area under peak) was measured for the Δ*orfH* mutant, CRISPRi strains, and vector control (pVect) strain, and plotted relative to the wild-type strain. The data are presented in the extracted ion chromatogram (Figure S4).

Following its discovery, lavendomycin was synthesized in vitro, but no biosynthetic mechanism has been reported.^54^ Using the BGC sequence and annotation from antiSMASH, we proposed a biosynthetic scheme for lavendomycin NRP (Table 1, Figure 9). The BGC encodes three multimodular NRPS genes (*lvdJ, lvdM, lvdO*) in addition to *lvdH*, which selects the starter unit, β-methylarginine, in the metabolite. Domain analysis using antiSMASH did not return a predicted substrate for the assembly line starter unit. Using PARAS, arginine was predicted to be the substrate.^55^ The lavendomycin-like BGC encodes conserved enzyme functions for a ketoarginine methyltransferase (*lvdA*) and a β-methylarginine biosynthesis bifunctional aminotransferase (*lvdG*) (Figure 9). A mechanism for β-methylarginine biosynthesis by *Streptomyces arginensis* has been reported involving the function of an aminotransferase and a SAM-dependent ketoarginine methyltransferase.^56,57^ Therefore, we hypothesized that the starter unit loads a β-methylarginine monomer. In addition, *lvdC* (argininosuccinate lyase) and *lvdE* (cysteine synthase/DABA synthase) were postulated to be involved in 2,3-diaminobutyric acid (DABA) biosynthesis through a modification of threonine.^58,59^ The DABA substrate is selected and incorporated by the first elongation module encoded by *lvdJ*. The downstream NRPS gene *lvdM* encodes two elongation modules. The first is selective for threonine, which is converted into dehydrobutyrine. The second selects serine. The *lvdM* gene also contains the only thioesterase (TE) domain associated with this cluster (Figure 9). The module organization indicates that LvdM carries out the final biosynthetic steps and terminates the biosynthesis despite its intermediate position in the operon. The *lvdO* gene encodes two modules that incorporate pipecolic acid and proline, with LvdL and LvdN supplying the substrates for the product biosynthesis (Table 1). The conversion of threonine into dehydrobutyrine and the function of *lvdD*, which encodes a kinase, remain to be determined.

**Figure 9:**
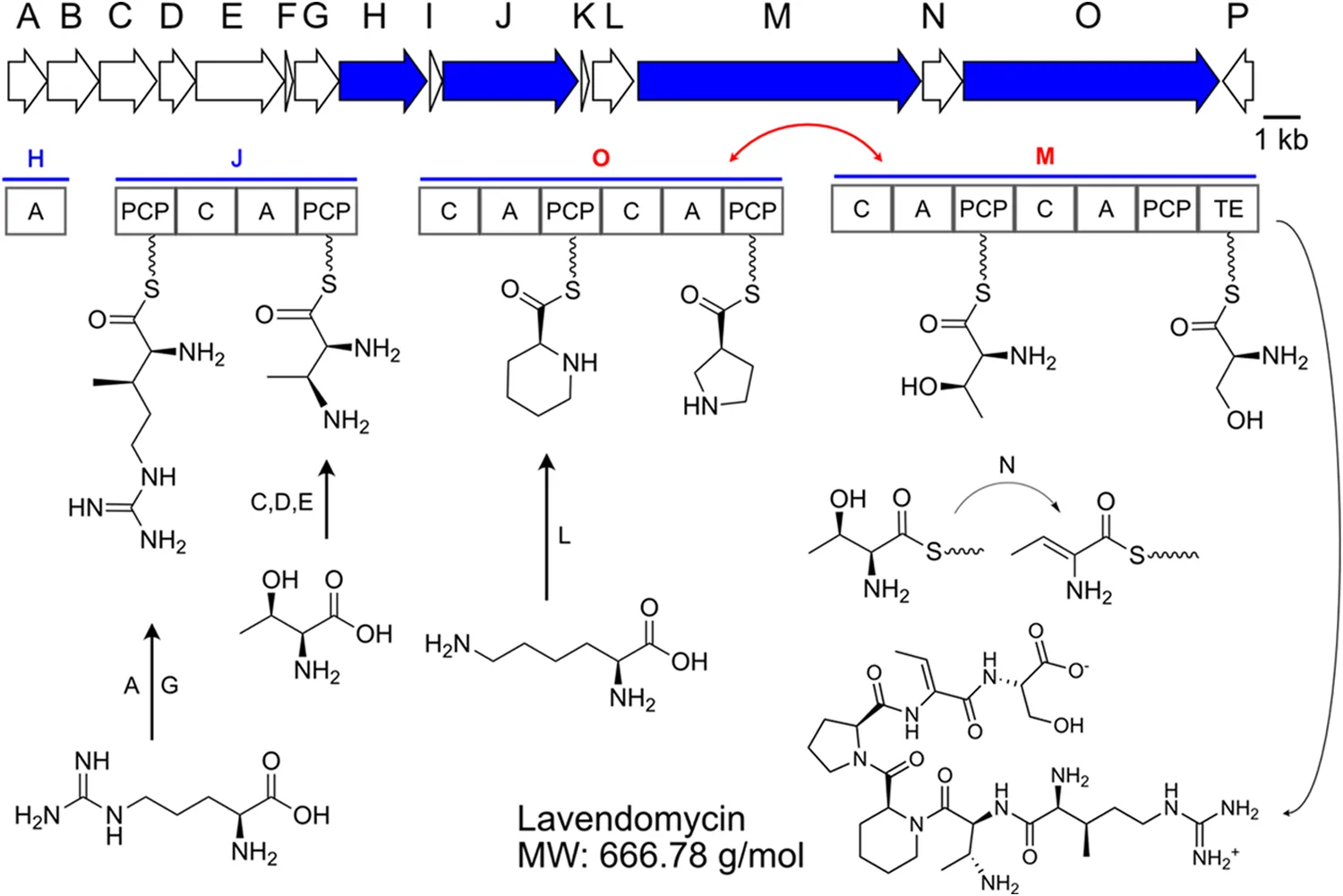
Proposed model for the biosynthesis of lavendomycin family of metabolites. Top: Schematic of the *lvd* BGC. **Bottom:** Substrate selectivity for individual enzymes and assembly line modules. LvdH selects β-methylarginine, a product of LvdA and LvdG methylation of arginine. LvdJ selects threonine or 2,3-diaminobutyric acid produced by LvdC–E. LvdO contains two modules. The first is pipecolic acid, produced by ornithine cyclodeaminase (LvdL). The second is proline. LvdM contains two modules and the only thioesterase encoded in the BGC. The first module selects threonine, which is converted to dehydrobutyrine (Dhb) by LvdN. The second module selects serine. The multimodular protein LvdM is proposed to function last in assembly with the TE domain, releasing the linear peptide. Table 1 shows the *lvd* gene-annotated gene functions.

**Table 1:** Deduced Functions of ORFs in the NRP Biosynthetic Gene Cluster

genes	proteins, corresponding to sequence similarity	proposed functions
A	Ketoarginine methyltransferase	Methylarginine synthesis
B	Transporter: Major Facilitator Superfamily	Transporter
C	Argininosuccinate lyase	2,3-Diaminobutyric acid synthesis
D	Protein kinase	2,3-Diaminobutyric acid synthesis
E	Cysteine synthase/DABA synthase	2,3-Diaminobutyric acid synthesis
F	MbtH-like protein	NRPS
G	β-methylarginine biosynthesis bifunctional aminotransferase	Methylarginine synthesis
H	NRPS	NRPS
I	MbtH-like protein	NRPS
J	NRPS	NRPS
K	MbtH-like protein	NRPS
L	Ornithine cyclodeaminase	Pipecolic acid synthesis
M	NRPS	NRPS
N	β-eliminating lyase	Dhb synthesis
O	NRPS	NRPS
P	Metallo-β-lactamase superfamily	Resistance

The order of genes in the *lvd* BGC is an uncommon example of a terminating thioesterase encoded in an upstream NRPS-encoding multimodular gene. To assess whether this gene order is conserved, we used Gator-GC^60^ to identify lavendomycin-like BGCs in the publicly available sequence databases. The output identified numerous examples of related BGCs that shared the same gene order and position of the TE domain as the *S*. Mg1 lavendomycin-like BGC (Figure S5). Therefore, we conclude that many actinomycetes encode the production of a family of peptides related to lavendomycin and that the organization of genes in the BGC is conserved. A detailed analysis of the biosynthetic mechanism and product structure is required to determine whether the final product is lavendomycin or whether the biosynthetic enzymes produce a variant form(s) of the NRP. These results demonstrate the utility of tandem-sgRNA CRISPRi as a reverse genetic approach to connect a BGC to a candidate metabolite.

## Discussion

In this study, we developed and used tandem-sgRNA CRISPRi as an efficient method to reveal loss-of-function phenotypes for *S.* Mg1 BGCs and their product metabolites. Initially, we found that CRISPRi, mediated by a single-guide RNA, only partially inhibited both the SfhA enzyme and linearmycins. We modified the pCZ2 vector to express two sgRNAs and found that this tandem-sgRNA CRISPRi provided substantial and stable knockdown of linearmycins. For each targeted BGC, we found that tandem-sgRNA CRISPRi produced substantially greater depletion than a single sgRNA for the product metabolite. Because a similar sgRNA configuration was effective for each BGC targeted in this study, we propose that tandem-sgRNA CRISPRi is an efficient method to disrupt secondary metabolite synthesis from many of the BGCs encoded in a selected *Streptomyces* genome. Loss of function frequently reveals developmental and competitive phenotypes of secondary metabolites. Thus, the resulting data provide a gateway to discover secondary metabolite functions and aid in prioritizing BGCs of unknown function for the identification of new secondary metabolites.

Although we have not determined a precise mechanism for tandem-sgRNA CRISPRi, the measurement of targeted transcripts for the *lnyHA* and *lvdA* genes indicates improved repression of transcription. The binding of dCas9 generally provides a roadblock to transcription, leading to the knockdown of targeted transcripts using CRISPRi.^30,61^ However, the impact of CRISPRi on a selected target can be incomplete, leading to partial or no discernible phenotypic changes. Some evidence indicates that the dCas9 complex may interfere with gene expression beyond providing a functional roadblock. Using HELA cells, CRISPRi decreased the concentrations of targeted proteins with no evident change in the corresponding mRNA levels.^62^ Thus, while a single sgRNA may suppress some targeted BGCs, our qPCR data for *lnyHA* and *lvdA* suggests that using two sgRNAs for a single promoter significantly improves transcriptional repression (Tables S5 and S6). Nevertheless, a precise mechanism for tandem sgRNAs will require further study. In yeast, rather than acting only as a roadblock, sgRNA/dCas9 binding creates an environment that is permissive for transcription initiation or termination, generating novel sense and antisense transcripts that ultimately interfere with successful transcription and translation of the target.^63^ Consistent with the previous results of CRISPRi in *Streptomyces* and other microbes, for *S.* Mg1, sgRNA(s) targeting the 5′ end of the transcript on the nontemplate strand resulted in the most reliable and effective repression.^61^ In each case, the tandem sgRNAs targeted dCas9 to sites near the −10 region of the 5′-UTR and near the translation start site (RBS) (Figure 10 and S6). Other configurations tested provided either partial or negligible knockdown. We propose that the two sgRNAs targeting the same 5′-UTR and RBS provide an effective block to transcription and possibly to translation initiation, culminating in a substantial reduction in the secondary metabolite biosynthesis. A distance greater than 30bp between each sgRNA is consistent with a model for the additive effects of two dCas9 complexes bound simultaneously, as opposed to sequential binding; however, the molecular mechanisms have yet to be investigated.^64,65^ We suggest that tandem-sgRNA CRISPRi may be effective for many BGCs and *Streptomyces* species, although some limitations remain. For example, in cases where the BGC is silent under the experimental conditions used, this approach may have limited utility. Also, we engineered the pCZ2-2 plasmid to insert into the genome for stable expression, necessitating a compatible phage attachment site for insertion. However, the plasmid can be engineered for insertional compatibility with many organisms or possibly maintained as a plasmid for the application of CRISPRi.

**Figure 10:**
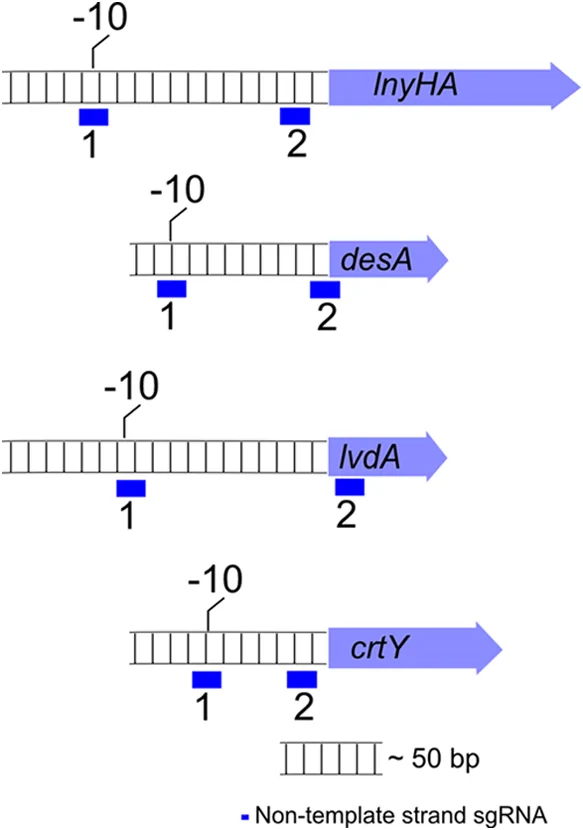
Summary of tandem-sgRNA CRISPRi targeting for BGC product knockdown. Among the sgRNA configurations tested, the most effective was two nontemplate strand, tandem sgRNAs that target a promoter within a selected BGC. For each of the four BGCs targeted in this study, the location of the tandem sgRNAs is depicted. An upstream sgRNA generally overlaps the −10 region of the promoter, and a downstream sgRNA is located near the ribosome-binding site (RBS) or start site for translation. The proximity of the tandem sgRNAs is variable, with ∼50 bases being the minimum distance tested that provided effective CRISPRi. The target gene clusters are as follows: *lnyHA*, linearmycins; *desA*, desferrioxamine; *lvdA*, lavendomycin; *crtY*, β-carotene.

We used the tandem-sgRNA CRISPRi in this study to illustrate its applicability to the secondary metabolome of a *Streptomyces* species. Many *Streptomyces* species produce carotenoids, but their physiological functions remain unclear. We applied tandem-sgRNA CRISPRi to identify the primary carotenoid product of the *crt* BGC as β-carotene. The CRISPRi strain enabled us to identify a phenotype associated with the loss of β-carotene. Intense fluorescence in both red and green channels was substantially depleted in the CRISPRi knockdown strain, while control strains using a single sgRNA did not sufficiently deplete the metabolite. This result highlights the utility of a facile method for engineering mutants in BGCs for diverse metabolites. Most secondary metabolites have no known function, primarily because they have not yet been identified and characterized. The ability to generate reliable disruptions is key to unveiling secondary metabolic functions and building knowledge of the physiological relevance of diverse metabolites.

The secondary metabolites of primary interest are those with antibiotic activity. In this study, we were able to disrupt an NRPS without a known product and observe a phenotype, leading to the identification of a lavendomycin-like metabolite and the assignment of its BGC. Although the antibiotic lavendomycin has been identified previously, a relevant BGC has not yet been identified. We suspect that the primary challenges in identifying its BGC are the incorporation of modified amino acids and the noncanonical arrangement of NRPS-encoding genes. Notably, the upstream gene, *lvdM*, encodes the only TE domain in NRPS and likely functions last in the assembly line biosynthesis of the lavendomycin-like metabolite. The downstream gene, *lvdO*, encodes intermediate assembly line enzymes for the incorporation of cyclized amino acid substrates. Therefore, our proposed biosynthetic order for enzyme function is based on the metabolite structure, reversing the order of LvdM and LvdO (Figure 9). Analysis using GATOR-GC revealed that this gene organization is conserved in species encoding BGC. It is unclear what the functional significance may be for this gene organization relative to the linear assembly line biosynthetic model. Nonetheless, the identification of a candidate lavendomycin BGC now opens the door to the analysis of its biosynthetic mechanism, candidate docking motifs, and order of enzymes in the biosynthetic complex, as well as for engineering variants of the antibiotic.

## Methods

### Strains and Growth Conditions

Strains and plasmids used in this study are listed in Table S3. We cultured *E. coli* strains at 37 °C in lysogeny broth (LB) [1% tryptone (Bacto), 0.5% yeast extract (BBL), 0.5% sodium chloride (Sigma)], or on LB agar plates [1.5% Agar (Bacto)]. *Streptomyces* strains were propagated in MYM [0.4% malt extract (Bacto), 0.4% yeast extract (BBL), and 0.4% D-(+)-maltose monohydrate (Sigma)] medium at 30 °C, unless otherwise indicated. Spore preparations were performed, as described by Keiser and Bibb.^66^ A lawn of *Streptomyces* was plated or streaked on agar plates containing MYM for *S*. Mg1 and R2YE for *S. coelicolor*, and incubated at 30 °C until thick spores were formed. Spores were collected from plates using 5 mm cell scrapers, resuspended in 1 mL sterile water, and stored at 4 °C. A typical spore prep contains around 10^8^∼10^10^ spores per milliliter, as determined by serial dilution plating and calculation based on CFU. To observe morphological differences between mutant strains and wild-type, we spotted 10 μL of 10^6^ or 10^7^ spores/mL of *Streptomyces* on MYM plates.

### Bioinformatic Platforms and Tools

We submitted the *S.* Mg1 (NZ_CP011664.1) genome sequence to antiSMASH to identify, annotate, and analyze secondary metabolite biosynthetic gene clusters in a genome-wide manner.^31^ Data were downloaded and further analyzed. NCBI databases were utilized to blast search for nucleotides or protein sequences. The BPROM online prediction tool (softberry.com) was used to predict −10 and −35 sequences for the 5′UTRs of the targeted BGCs. The downstream sgRNA was selected based on its relative proximity to the transcription start site and was thus designated as the RBS.

### Construction of CRISPRi Shuttle Plasmids

All DNA manipulations were carried out using *Escherichia coli* DH5α. The primers and protospacers (sgRNAs) used in this study are listed in Table S4. The *rpsL* promoter, Cas9, and protospacer cassette (including *gapdH* promoter, *lacZ*, tracrRNA, and terminator) were adopted from pCRISPomyces-2 plasmids.^36^ Two nonsynonymous mutations, D10A and H840A, were incorporated to deactivate the Cas9 cleavage activity. The DNA fragment was synthesized by IDT Synthetic gBlocks, comprising the *rpsL* promoter sequence minus the *Bsa*I restriction site and point mutation D10A (GAC→GCC). We assembled the synthesized DNA fragment, amplified dCas9 with point mutation H840A (CAC→GCC), and protospacer cassette from pCRISPomyces-2, with an integration plasmid backbone pSET152. The finalized CRISPRi integration plasmid, pCZ2, carried a φC31 *attP* site and a gene that encoded for integrase. The protospacer of a target cluster was first inserted via *Bsa*I-mediated golden gate assembly, as previously described.^67^ The second protospacer cassette was subsequently inserted by Gibson assembly using an *Xba*I linearized construct containing the first protospacer cassette.

### Construction of CRISPRi Strains

The 20-nucleotide protospacer of a target cluster was first inserted into the pCZ2 vector via *Bsa*I-mediated golden gate assembly and selected on LB plates containing 50 μg/mL apramycin, 0.8 mM IPTG, and 40 μg/mL β-D-galactopyranoside (X-gal). The second protospacer cassette, including the gapdh promoter, sgRNA, tracrRNA, and terminator, was subsequently inserted by Gibson assembly using an *Xba*I linearized construct containing the first protospacer cassette. The constructs were verified by PCR amplification with primers listed in Table S4 and sequencing alignments.

### Interspecies Conjugation

Confirmed recombinant constructs containing either single-guide RNA or double-guide RNAs were transformed into *E. coli* ET12567. A single colony was propagated in liquid LB media containing apramycin (30 μg/mL) and chloramphenicol (5 μg/mL) to an OD_600_ of ∼0.4. The cells were pelleted and washed with LB without antibiotics. Equal volumes of *E. coli* (OD_600_ ∼ 0.4) cells were mixed with 10^6^, 10^7^, and 10^8^ per milliliter of *Streptomyces* spores and spotted on AS1 [0.1% yeast extract, 0.5% soluble starch, 0.25% NaCl, 1% Na_2_SO_4_, pH adjusted to 7.5 with KOH, supplemented with 0.02% l-alanine, 0.02% l-arginine, 0.05% l-asparagine, and MgCl_2_ after autoclave] agar plates. A negative control with only *Streptomyces* spores was also spotted on the plates. After 16 h of incubation at 30 °C, each plate was flooded with 1.5 mL of antibiotic solution to obtain final concentrations of 50 μg/mL apramycin and 30 μg/mL nalidixic acid. After drying, the plates were incubated at 30 °C for 2–4 days with continuous monitoring.

### Extraction and Detection of Linearmycins from S. Mg1 Strains

Buffered MYM liquid media (50 mL in 250 mL Erlenmeyer flasks) were inoculated with 10^5^ spores of *S*. Mg1 from a freshly prepared spore suspension. The cultures were incubated at 30 °C in the dark and shaken at 250 rpm for 2 days. All linearmycin extraction and purification procedures were carried out in the dark as described previously.^38^ *S*. Mg1 mycelia were collected by centrifugation. The mycelial pellet of each sample was extracted sequentially with 50 mL of methanol and 50 mL of ethanol at room temperature and shaken at 200 rpm for 30 min. The extracts were then combined and dried in a rotary evaporator. The residues were dissolved in 200 μL of 85% methanol. Insoluble precipitates were removed by centrifugation. The supernatants were transferred to screw-cap vials for storage at −20 °C until use.

Linearmycin analysis was performed using an Agilent 1200 HPLC system. A 10 μL volume of each sample was injected onto a Luna C18(2) column (4.6 × 250 mm, 5 μm; Phenomenex). The run was performed at a flow rate of 1.0 mL/min at 30 °C. The samples were eluted with mobile phases A (acetonitrile) and B (0.1% (v/v) formic acid). The elution gradient was initiated with 40% A and 60% B and reached 50% A and 50% B over 10 min. A 5 min step with 75% A and 25% B was maintained for linearmycin elution. The column was equilibrated with 40% A and 60% B for 5 min. Linearmycin peaks were detected at 333 nm as previously reported.^38^ We confirmed the specificity of linearmycin by comparing the HPLC peaks from *S.* Mg1 wild-type and the Δ*lnyI* strain (abolished linearmycin biosynthesis) and by MALDI-TOF.

### Extraction and Detection of Desferrioxamine from S. Mg1 Strains

50 mL of semisynthetic GG1 liquid media, supplemented with 200 μM of 2′,2′-dipyridyl for iron deficiency, was inoculated with 10^5^ spores of *S*. Mg1 strains from a freshly prepared spore suspension. The cultures were incubated at 30 °C for 4 days while shaking at 250 rpm. *S*. Mg1 mycelia were removed by centrifugation. The supernatants were lyophilized to dryness and redissolved in 200 μL of 50% methanol. A 40 μL sample of each was mixed with 1 μL of 200 mM ferric chloride for the chelation of desferrioxamine before HPLC detection. For quantitative analysis, samples (10 μL) were eluted with a gradient of 10% acetonitrile (A) and 90% 0.1% (v/v) formic acid (B), which reached 40% A and 60% B after 5 min. A 2 min 90% A, 10% B step was used for washing the crude extracts. The column was equilibrated with 10% A and 90% B for 3 min. The desferrioxamine peak was monitored at a 430 nm wavelength. The quantification for each sample was performed by integrating the area under the relevant peaks on the elution chromatography.

### Extraction and Detection of Carotenoids

Carotenoid extraction was carried out using the protocol described by Myronovskyi et al.^68^ 50 mL of MYM7 media was inoculated with spores of different strains of *S*. Mg1 (10^7^ spores/mL) and cultivated under aeration at 30̊C for 3 days. After centrifugation, carotenoids were extracted from the supernatant using chloroform and from the mycelium using chloroform:methanol (1:1). The chloroform extracts were evaporated and resuspended in chloroform for LC-MS analysis. The crude extracts of carotenoids were loaded onto a ProntoSIL 200–5-C30 column for HPLC analysis. Chromatographic separation was carried out on Ultimate 3000 HPLC (Thermo Fisher) using mobile phase B (10 mM ammonium formate in 9:1 v/v isopropanol:acetonitrile) with 0.1% formic acid and mobile phase A (10 mM ammonium formate in 6:4 v/v acetonitrile:water) with 0.1% formic acid. The gradient was set from 70% to 100% mobile phase B with a 0.8 mL/min flow rate. Carotenoids were monitored using a Diode Array Detector (DAD) signal at 430 nm. Pure β-carotene (SIGMA, USA) was used as the control for LC-MS. The carotenoids were identified by mass spectrometry (Thermo Scientific QE Focus). Ionization method: ESI, Sheath gas flow: 60, Aux gas flow: 20, Spray voltage: 3.5 kV, Capillary temp: 38 °C, S-lens RF level: 50, Aux gas heater temp: 300 °C.^69,70^

### Imaging of Carotenoid-Producing and Mutant Strains

Two-day-old cultures (1 mL) of vector control, single sgRNA, and dual-sgRNA *S*. Mg1 strains were centrifuged, and the pellets were resuspended in 100 mL of water. 3 mL of the resuspended culture was spotted and spread using a coverslip. Imaging of the intrinsic fluorescence from S. Mg1 strains was carried out using a Lionheart FX automated microscope (Agilent) with a 100× oil immersion lens. Brightfield images were acquired with a 50 ms (ms) exposure, while fluorescence images were captured using a GFP filter (Ex: 469 nm; Em: 525 nm) and propidium iodide filter (Ex:531; Em: 647 nm) acquired with a 500 ms exposure. Multiple images from different areas of the slides were acquired at random. Upon acquisition, the intensities of all of the images were normalized to the intensity of the vector control *S*. Mg1 strain using the Fiji-ImageJ software.

### Extraction and Detection of Lavendomycin

Buffered MYM7 liquid media (50 mL in 250 mL Erlenmeyer flasks) was inoculated with 10^5^ spores of *S*. Mg1 from a freshly prepared spore suspension. The cultures were incubated at 30 °C with shaking at 250 rpm for 5 days with around 20 glass beads to prevent aggregation. We removed *S.* Mg1 mycelia by centrifugation. The supernatants were cleaned with equal volumes of ethyl acetate in separatory funnels. The aqueous phases were collected and lyophilized. The residues were redissolved in 200 μL of 50% methanol, and insoluble precipitates were removed by centrifugation. The supernatants were transferred to screw-cap vials for storage or experiments.

To identify the NRP and measure the metabolic differences between the mutant strains and the wild-type, we spotted 1 μL of diluted crude extracts from *Streptomyces* (10-fold dilution with 50% acetonitrile and 50% methanol) in a well of MALDI plates. The samples were air-dried after overlaying with 1 μL of α-cyano-4-hydroxycinnamic acid MALDI matrix. Spectra were collected using a Bruker Ultraflextreme MALDI-TOF/TOF mass spectrometer in the positive mode for detection and analysis.

For quantification, buffered MYM7 agar plates (25 mL per plate; four plates per strain) were inoculated with 100 μL of freshly prepared *S*. Mg1 spore suspension (10^6^ spores/mL) and incubated at 30 °C for 48 h. The agar was cut into 0.5 cm cubes, frozen at −80 °C until solid, and then thawed prior to extraction. Mycelial debris and agar were removed through centrifugation. The resulting supernatants were extracted with an equal volume of ethyl acetate using separatory funnels. The aqueous phases were collected, lyophilized, and resuspended in 200 μL of 50% methanol. Insoluble material was removed by centrifugation, and the supernatants were transferred to screw-cap vials for storage or downstream analysis.

### Untargeted Liquid Chromatography High-Resolution Mass Spectrometry (LC-HRMS)

Untargeted liquid chromatography high-resolution mass spectrometry (LC-HRMS) analysis was performed on a Q Exactive Plus Orbitrap mass spectrometer (Thermo Scientific, Waltham, MA) coupled to a binary pump HPLC (UltiMate 3000, Thermo Scientific). Full MS was obtained at a 70,000 resolution (200 *m*/*z*) with a scan range of 133–2000 *m*/*z*. Full MS followed by ddMS2 spectra were obtained at 35,000 and 17,500 resolutions (200 *m*/*z*) with a 1.5 *m*/*z* isolation window and stepped NCE (20, 40, 60). The samples were maintained at 4 °C before injection. The injection volume was 10 μL. Chromatographic separation was achieved on a Synergi Fusion 4 μm, 150 mm × 2 mm reverse phase column (Phenomenex, Torrance, CA) maintained at 30 °C using a solvent gradient method. Solvent A was water (0.1% formic acid). Solvent B was methanol (0.1% formic acid). The gradient method used was 0–5 min (10% B to 40% B), 5–7 min (40% B to 95% B), 7–9 min (95% B), 9–9.1 min (95% B to 10% B), and 9.1–13 min (10% B). The flow rate was 0.4 mL/min. Sample acquisition was performed with Xcalibur (Thermo Scientific). To analyze the data generated from LC-MS, we used the R statistical programming language. Raw data containing both MS and MS/MS spectra was converted into mzXML format before being imported for a pairwise comparison. After peak detection, retention time correction, and peak grouping across samples using “xcms” and “faahKO” packages from Bioconductor, the output was processed and visualized using “dplyr” and “ggplot2” packages.

## Supplementary Material



## Notes

HPLC and biological assays, mass spectrometry, and output of genome analyses and sequence (Figures S1–S6); predicted BGCs, standard CRISPR outcomes, strains, and primers used (Tables S1–S4); qRT-PCR results (Tables S5–S6) (PDF)
