# Supplementary material for: Tandem-sgRNA Provides an Effective Reverse Genetic Approach for Suppression of Streptomyces Biosynthetic Gene Clusters and Secondary Metabolism
Source: ACS Synth Biol. 2026 Jul 11;15(8):3382–95. doi: 10.1021/acssynbio.6c00274 (PMC13505331; doi:10.1021/acssynbio.6c00274)
Supplement: Supplementary file 1 [file sb6c00274_si_001.pdf]

Supporting Information for Publication

Title: Tandem-sgRNA provides an effective reverse-genetic approach for suppression of *Streptomyces* biosynthetic gene clusters and secondary metabolism.

Authors: Chengxi Zhang[1], Nitya Kalyani Josyula[1], Ivette Cornejo-Corona[1], Rebecca Watson[1], José D. Cediel-Becerra[3], Marc G. Chevrette[4], Timothy P. Devarenne[1], Paul D. Straight[1,2]

Affiliations: [1]Department of Biochemistry & Biophysics and [2]Graduate Program in Genetics and Genomics, Texas A&M University, AgriLife, College Station, TX, 77843, United States. [3]Department of Microbiology and Cell Science, University of Florida, Gainesville, FL, 32611, United States. [4]Department of Plant Pathology and Wisconsin Institute for Discovery, University of Wisconsin-Madison, WI, 53706, United States.

To whom correspondence may be addressed. Email: paul_straight@tamu.edu.

Supplemental Figures, Tables, Methods, and References.

Supplemental Figures:

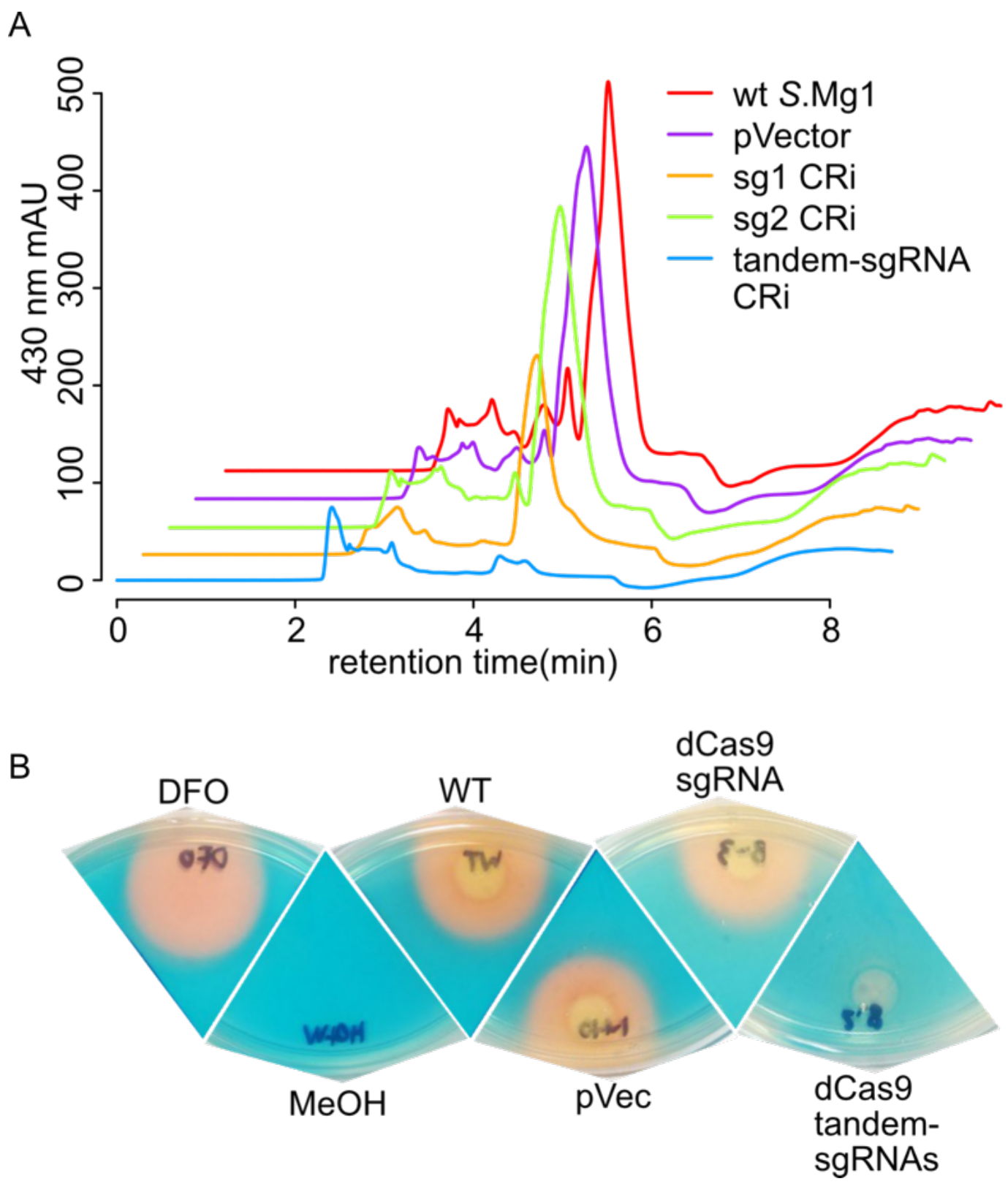

Figure S1. Depletion of desferrioxamines using tandem sgRNA-CRISPRi. **A.** HPLC chromatogram of desferrioxamine extracts from *S.* Mg1 strains. Desferrioxamines were detected at 430 nm. The data was used for quantitation of desferrioxamine shown in Figure 5. **B.** A chrome azurol S (CAS) assay to detect the presence of desferrioxamines in culture media. Extracts from *S.* Mg1 strains in 50% methanol were deposited on agar media containing the CAS dye. Desferrioxamines sequester iron, depleting the blue pigmentation. Samples from left to right are purified desferrioxamine B (Sigma-Aldrich) and 50% methanol controls, followed by extracts from: wild-type *S.* Mg1, vector control *S.* Mg1, a single sgRNA *S.* Mg1 strain, and the tandem-sgRNA CRISPRi strain. The tandem sgRNA strain disrupted desferrioxamine production. The assay was carried out according to the protocol in Schwyn and Neilands, 1987 [1].

A

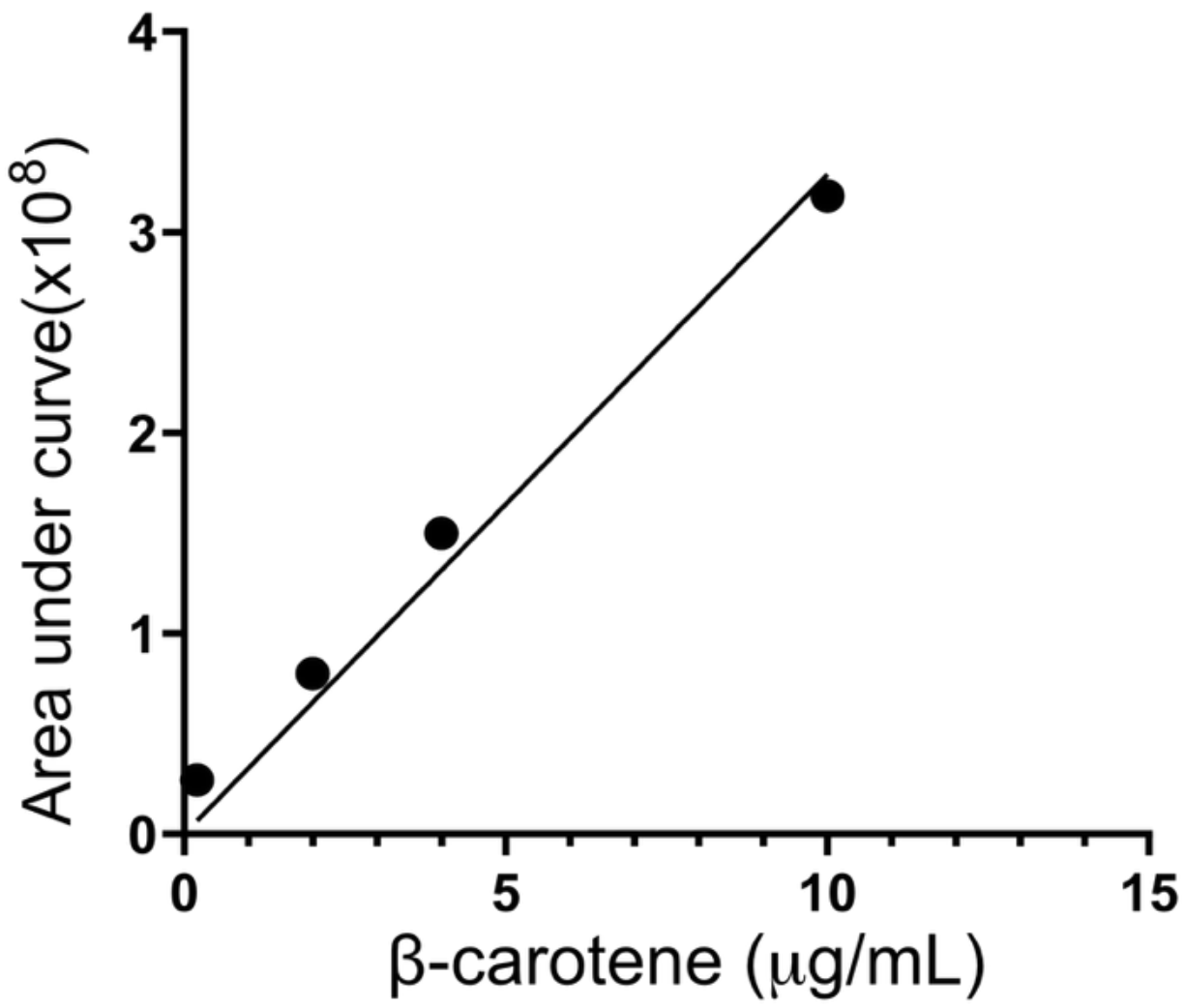

B

Mass fragments from β-carotene standard	Mass fragments from sample	Expected fragments for lycopene *	Mass fragments from sample
520.54	520.58	494.9	495.03
476.34	476.32	467.37	467.31
281.22	281.27	343.24	343.235
268.29	268.31	171.12	171.24
210.08	210.16	137.132	137.096
137.13	137.09	123.118	123.117
123.11	123.08		

* Mass fragments for lycopene obtained from https://massbank.eu/MassBank/RecordDisplay?id=MSBNK-MSSJ-MSJ00130

Figure S2. *S.* Mg1 β-carotene identification and quantitation. A. LC-MS ion intensities from serial dilution of pure β-carotene were used to generate a standard curve for quantitative mass spectrometry of β-carotene produced by *S.* Mg1 strains. **B.** Mass fragments identified for β-carotene and lycopene produced by wild-type and *crtY*-targeted, tandem-sgRNA CRISPRi depleted *S.* Mg1 strains, respectively. For β-carotene, pure sample was used to generate standard (expected) ions. For lycopene, expected fragment ion masses were obtained from MassBank.

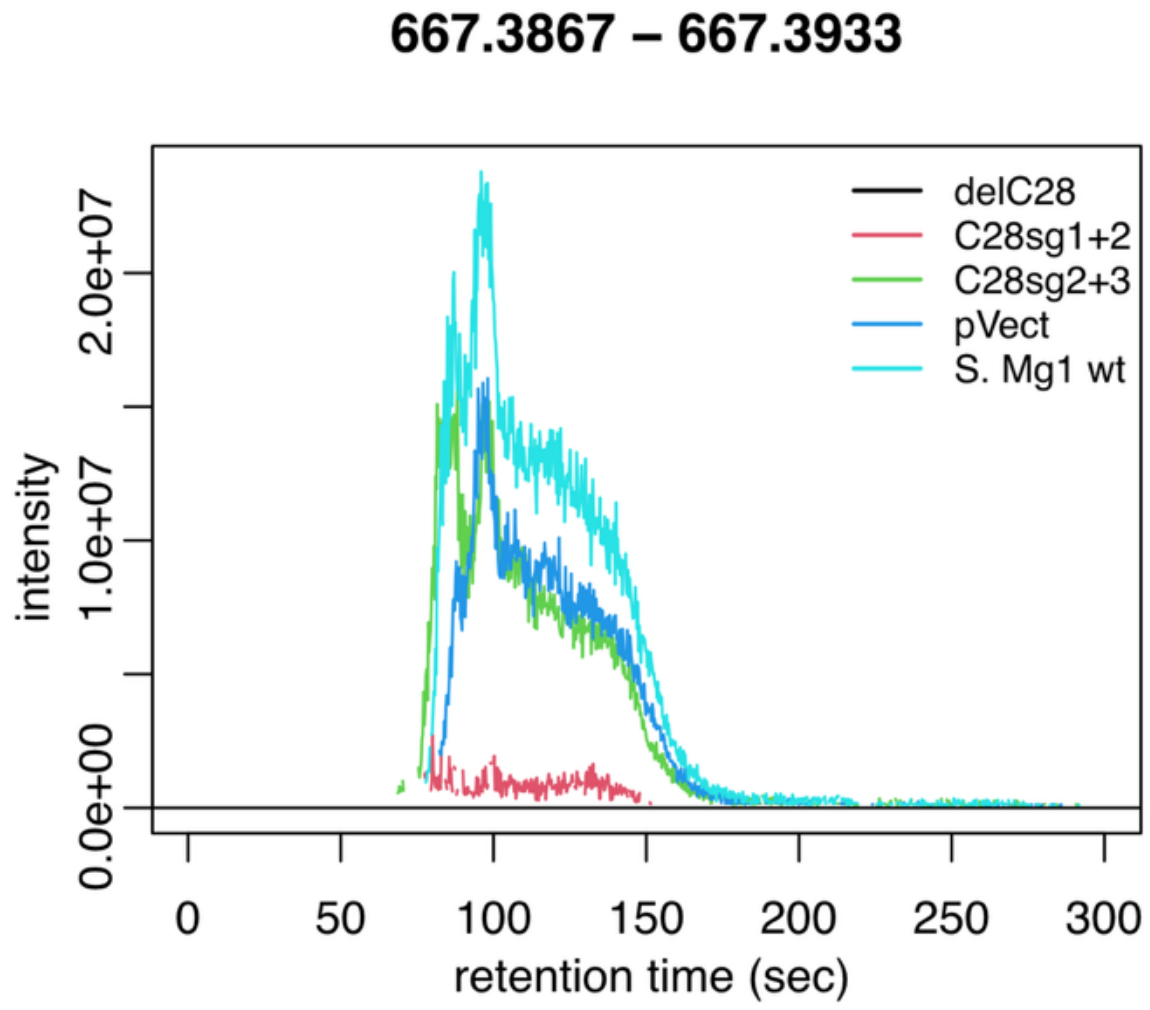

Figure S3. Extracted ion chromatogram of Cluster 28 NRP from LC-MS. Ion intensity in the indicated mass range (*m/z* = 667.3867-667.3933) for extracts from wild-type *S.* Mg1, vector control *S.* Mg1, Δ*orfH*, and *orfA*-targeting tandem-sgRNA CRISPRi strains, sg1+2 and sg2+3, respectively. The samples were prepared identically for comparison of relative amounts of the product peptide in extracts from each strain.

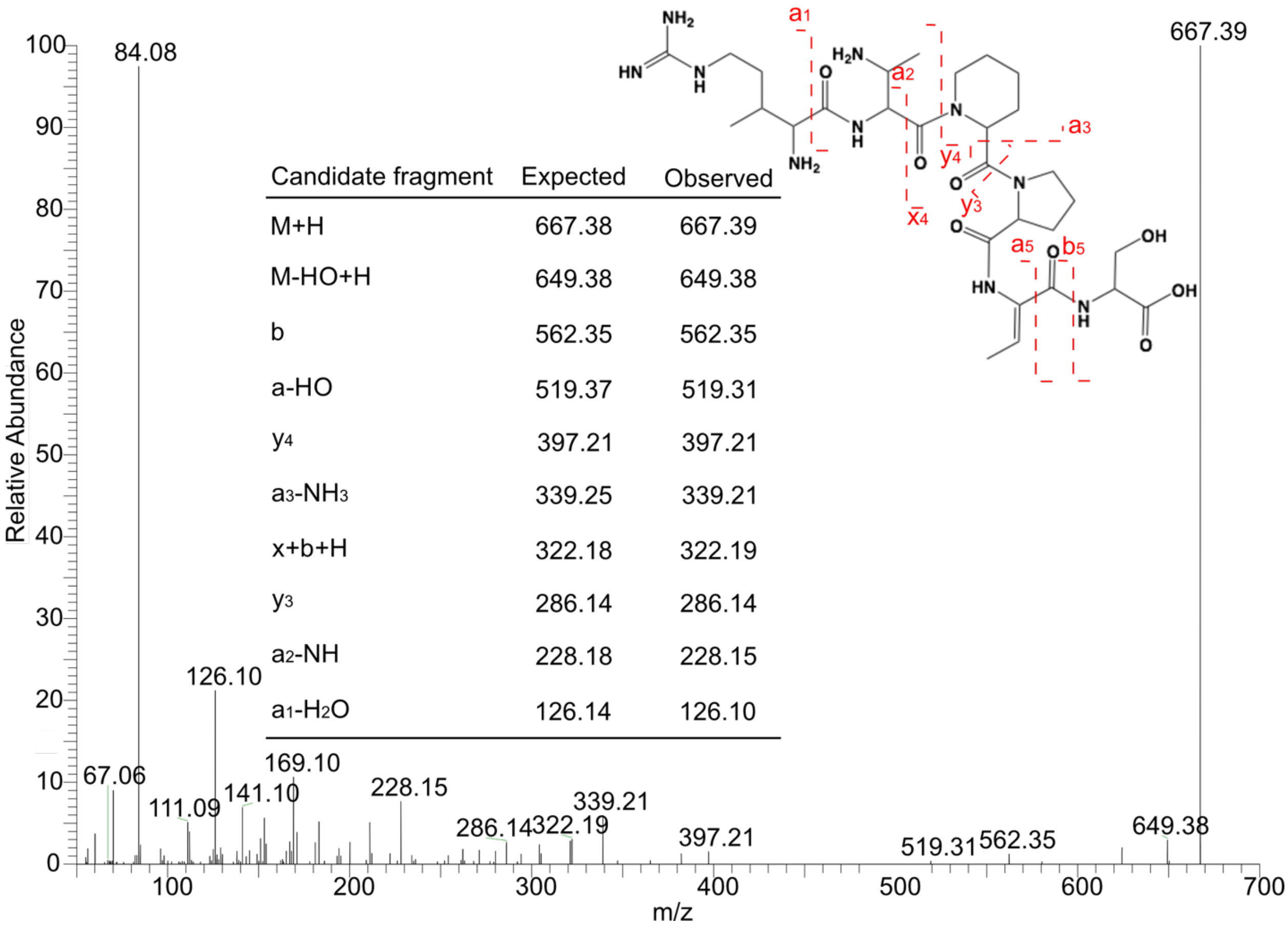

Candidate fragment	Expected	Observed
M+H	667.38	667.39
M-HO+H	649.38	649.38
b	562.35	562.35
a-HO	519.37	519.31
y_4	397.21	397.21
a_3-NH_3	339.25	339.21
x+b+H	322.18	322.19
y_3	286.14	286.14
a_2-NH	228.18	228.15
a_1-H_2O	126.14	126.10

Figure S4. Confirmation of the lavendomycin-like peptide using LC-MS/MS. LC-MS fragmentation spectrum of the Cluster 28 parent ion, *m/z* 667.39. Observed fragments match expected fragment ions match those of lavendomycin [2]. The inset shows the lavendomycin structure and associated fragmentation sites for the ions detected.

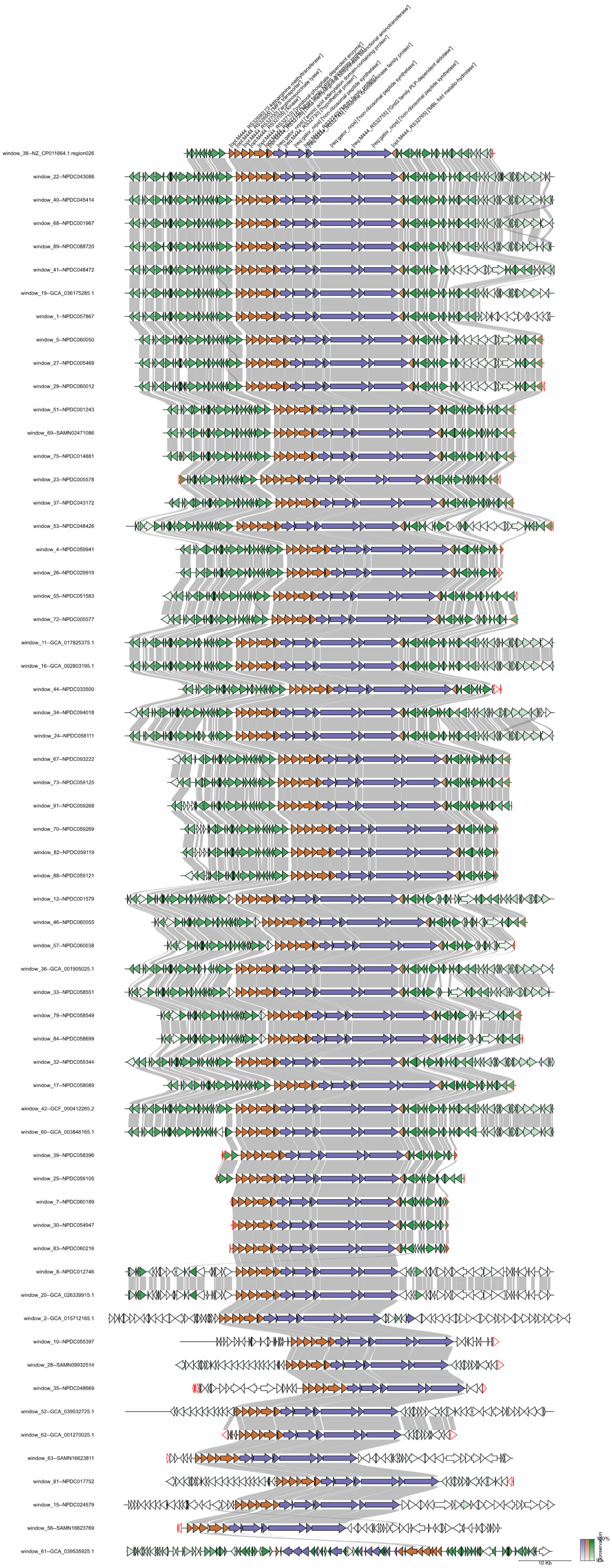
[opt:M444_RS32690] ['2-ketoarginine methyltransferase']
[opt:M444_RS32695] ['MFS transporter']
[opt:M444_RS32700] ['argininosuccinate lyase']
[opt:M444_RS32705] ['kinase']
[opt:M444_RS32710] ['pyridoxal-phosphate dependent enzyme']
[opt:M444_RS32715] ['MbtH family NRPS accessory protein']
[opt:M444_RS32720] ['beta-methylarginine biosynthesis bifunctional aminotransferase']
[req:gator_nrps] ['amino acid adenylation domain-containing protein']
[req:M444_RS32730] ['hypothetical protein']
[req:gator_nrps] ['non-ribosomal peptide synthetase']
[req:M444_RS32740] ['MbtH family protein']
[req:M444_RS32745] ['ornithine cyclodeaminase family protein']
[req:gator_nrps] ['non-ribosomal peptide synthetase']
[req:M444_RS32755] ['GntG family PLP-dependent aldolase']
[req:gator_nrps] ['non-ribosomal peptide synthetase']
[opt:M444_RS32765] ['MBL fold metallo-hydrolase']
window_38--NZ_CP011664.1.region026
window_22--NPDC043088
window_40--NPDC045414
window_68--NPDC001967
window_89--NPDC088720
window_41--NPDC048472
window_19--GCA_036175285.1
window_1--NPDC057867
window_5--NPDC060050
window_27--NPDC005469
window_29--NPDC060012
window_51--NPDC001243
window_69--SAMN02471086
window_75--NPDC014881
window_23--NPDC005578
window_37--NPDC043172
window_53--NPDC048426
window_4--NPDC059941
window_26--NPDC029919
window_55--NPDC051583
window_72--NPDC005577
window_11--GCA_017825375.1
window_16--GCA_002803195.1
window_44--NPDC033500
window_34--NPDC094018
window_24--NPDC058111
window_67--NPDC093222
window_73--NPDC059125
window_91--NPDC059268
window_70--NPDC059269
window_82--NPDC059119
window_88--NPDC059121
window_12--NPDC001579
window_46--NPDC060055
window_57--NPDC060038
window_36--GCA_001905025.1
window_33--NPDC058551
window_79--NPDC058549
window_84--NPDC058699
window_32--NPDC059344
window_17--NPDC058089
window_42--GCF_000412265.2
window_60--GCA_003846165.1
window_39--NPDC058396
window_25--NPDC059105
window_7--NPDC060189
window_30--NPDC054947
window_83--NPDC060216
window_8--NPDC012746
window_20--GCA_026339915.1
window_2--GCA_015712165.1
window_10--NPDC055397
window_28--SAMN09932514
window_35--NPDC048669
window_52--GCA_039532725.1
window_62--GCA_001270025.1
window_63--SAMN16623811
window_81--NPDC017752
window_15--NPDC024579
window_56--SAMN16623769
window_61--GCA_039535925.1
10 Kb
100%
conservation
0%

Figure S5. Gator-GC output. Neighborhoods window depicting gene conservation and order relative to lavendomycin BGC input [3]. Zoom image to view gene order and sequence identifiers. The lavendomycin BGC is conserved across many *Strepomyces* species, preserving the gene order found in *S.* Mg1.

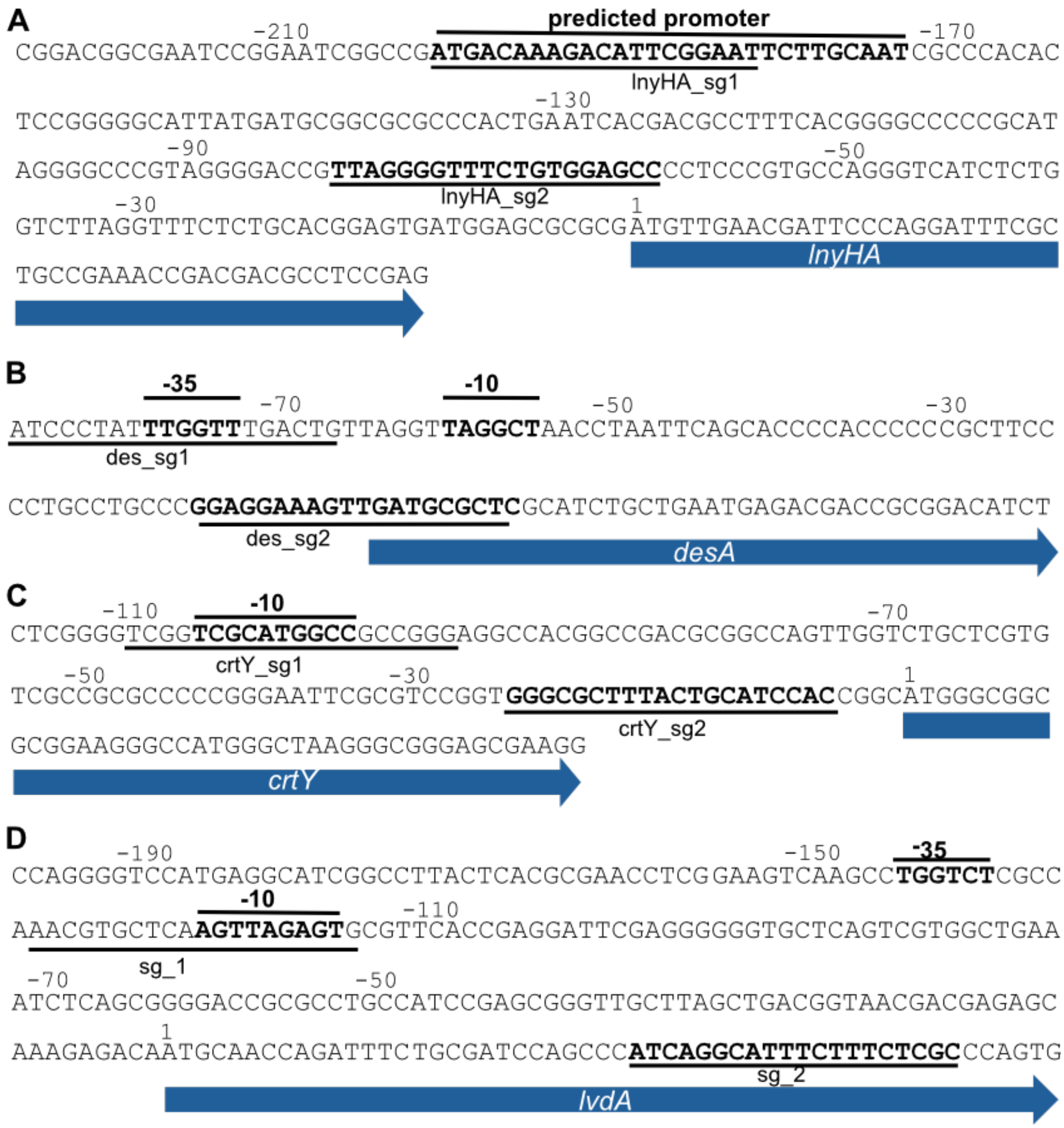

Figure S6. Upstream regions for each BGC indicating location of sgRNA-targeted sequences. 5’UTR sequences for the biosynthetic gene clusters for **A**- linearmycins; **B**- deferoxamine; **C**- β-carotene and **D**- lavendomycin. The -10 and -35 sequences are highlighted for deferoxamine, carotenoid and lavendomycin sequences. The predicted promoter site for linearmycin BGC is more ambiguous with -35 and -10 sequences unresolved. Sequences nearer to the translation start site -labeled rbs for ribosome-binding sites, are indicated by the position of the second sgRNA used, respectively.

Supplemental Tables:

Table S1. AntiSMASH analyses of *Streptomyces* sp. Mg1 (NCBI reference sequence: NZ_CP011664.1[69]).

Cluster	Type	From	To	Most similar known cluster (Similarity)
Cluster 1	nucleoside	30,256	51,167	pentostatine/vidarabine (6%)
Cluster 2	terpene	127,319	152,797	isorenieratene (100%)
Cluster 3	NRPS-like, T1PKS	501,941	550,385	neoabyssomicin/abyssomicin (6%)
Cluster 4	NRPS, terpene	554,742	615,429	bohemamine A/bohemamine B/bohemamine C (12%)
Cluster 5	lanthipeptide-class-iii, terpene	745,744	772,860	
Cluster 6	NRPS, T1PKS, NAPAA, transAT-PKS, terpene	932,552	1,051,615	weishanmycin (100%)
Cluster 7	T1PKS	1,057,302	1,248,574	linearmycin A/linearmycin B/linearmycin C/linearmycin C (100%)
Cluster 8	RRE-containing, LAP, thiopeptide	1,268,239	1,302,825	glycinocin A (4%)
Cluster 9	hglE-KS, T1PKS, terpene	1,637,598	1,696,261	hexacosalactone A (9%)
Cluster 10	lassopeptide	2,448,305	2,470,637	
Cluster 11	NI-siderophore	3,136,817	3,166,598	desferrioxamine B (100%)
Cluster 12	lanthipeptide-class-i	3,564,824	3,589,493	skyllamycin D/skyllamycin E (2%)
Cluster 13	lanthipeptide-class-i	4,292,765	4,317,328	
Cluster 14	lanthipeptide-class-i	5,105,224	5,130,687	chejuenolide A/chejuenolide B (7%)
Cluster 15	terpene-precursor	5,264,140	5,285,213	
Cluster 16	NI-siderophore	5,839,455	5,872,480	schizokinen (20%)
Cluster 17	Hydrogen-cyanide synthase	6,036,831	6,049,808	Aborycin (21%)

Cluster 18	RiPP-like	6,103,526	6,114,881	
Cluster 19	terpene	6,180,567	6,202,783	toxoflavin/fervenulin (10%)
Cluster 20	terpene	6,479,894	6,506,775	hopene (61%)
Cluster 21	lanthipeptide-class-iii, lanthipeptide-class-ii	6,631,172	6,657,028	
Cluster 22	T1PKS	6,879,019	6,958,618	aldgamycin J/aldgamycin K/aldgamycin P/aldgamycin E (68%)
Cluster 23	lanthipeptide-class-iii	6,990,661	7,013,297	SapB (100%)
Cluster 24	melanin	7,093,620	7,121,271	melanin (28%)
Cluster 25	NI-siderophore, T3PKS, T2PKS	7,180,328	7,292,065	spore pigment (66%)
Cluster 26	terpene	7,293,753	7,314,790	
Cluster 27	terpene-precursor	7,320,153	7,341,277	
Cluster 28	NRPS	7,511,748	7,571,753	leupeptin Pr/leupeptin Ac (50%)

Previously observed compounds include linearmycins (cluster 7), chalcomycins (cluster 22).

Table S2. BGC editing result using pCRISPomyces-2 system in *S.* Mg1

**Cluster**	**Type**	**Most similar known cluster (similarity)**	**Editing efficiency (edited/total exconjugants*)**
Cluster 3	NRPS-like, T1PKS	neoabyssomicin/abyssomicin (6%)	1/6
lny cluster	T1PKS	linearmycins (100%)	0/4
Cluster 9	hglE-KS, T1PKS, terpene	hexacosalactone A (9%)	0/0
Cluster 14	lantipeptide	chejuenolides (7%)	3/4
Cluster 28	NRPS	leupeptin Pr/leupeptin Ac (50%)	1/12

Predicted BGCs are designated with numbers based on their chromosomal loci. *The number of total exconjugants recovered from multiple conjugation experiments.

Table S3. Bacterial Strains

**Strains**	**Description**	**Source**
PDS0506	*Esherichia coli* DH5α	Laboratory collection
PDS0002	*Esherichia coli* ET12567	Laboratory collection
PDS0061	*Streptomyces coelicolor*	Laboratory collection
PDS0543	*Streptomyces* sp. Mg1	Laboratory collection
PSK0524	*Streptomyces sviceus*	Laboratory collection
PDS0066	NCIB3610 undomesticated *Bacillus subtilis*	Laboratory collection
PDS0930	*S. coelicolor attB*::pCZ2 Ω(lacZ::*redD* sgRNA)	This study
PDS0928	*S. coelicolor attB*::pCZ2 Ω(lacZ:: P*redD* sgRNA)	This study
PDS0927	*S. coelicolor attB*::pCZ2 Ω(lacZ:: *act*I-*ORF*1 sgRNA)	This study
PDS0929	*S. coelicolor attB*::pCZ2 Ω(lacZ:: *act*II-ORF4 sgRNA)	This study
PDS0997	*S.* Mg1 *attB*::pCZ2	This study
PDS0998	*S.* Mg1 *attB*::pCZ2 Ω(lacZ::*lnyI* sgRNA)	This study

PDS0999	*S.* Mg1 *attB*::pCZ2 Ω(lacZ:: *lnyHA*-sg1 sgRNA)	This study
PDS1000	*S.* Mg1 *attB*::pCZ2 Ω(lacZ:: *lnyHA*-sg2 sgRNA)	This study
PDS1001	*S.* Mg1 *attB*::pCZ2 Ω(lacZ:: *lnyHA*-sg1 *lnyI* sgRNA)	This study
PDS1002	*S.* Mg1 *attB*::pCZ2 Ω(lacZ:: *lnyHA*-sg1 *lnyHA*-sg2 sgRNA)	This study
PDS1003	*S.* Mg1 *attB*::pCZ2 Ω(lacZ:: *desA*-sg1 sgRNA)	This study
PDS1004	*S.* Mg1 *attB*::pCZ2 Ω(lacZ:: *desA*-sg2 sgRNA)	This study
PDS1005	*S.* Mg1 *attB*::pCZ2 Ω(lacZ:: *desA*-sg1 *desA*-sg2 sgRNAs)	This study
PDS0830	*S.* Mg1 cluster 3 frameshift deletion	This study
PDS0839	*S.* Mg1 ΔM444_22370 in cluster 14	This study
PDS0837	*S.* Mg1 frameshift deletion in cluster 28	This study
PDS1006	*S.* Mg1 *lnyI* frameshift deletion	This study
PDS1264	*S.* Mg1 *attB::*pCZ2 Ω(lacZ:: ORF-A-sg 1+sg2)	This study
PDS1265	*S.* Mg1 *attB::*pCZ2 Ω(lacZ:: ORF-A -sg 1+sg3)	This study
PDS1266	*S.* Mg1 *attB::*pCZ2 Ω(lacZ:: ORF-A-sg2+sg3)	This study
PDS1328	*S.* Mg1 *attB*::pCZ2 Ω(*lacZ*::*crtY*sgRNA1)	This study
PDS1329	*S.* Mg1 *attB*::pCZ2 Ω(*lacZ*::*crtY*sgRNA2)	This study
PDS1330	*S.* Mg1 *attB*::pCZ2 Ω(*lacZ*::*crtY*sgRNA1+sgRNA2)	This study
**Plasmids**	**Description**	**Source**
pCRISPomyces-2	Apr^{R}, *oriT*, $rep^{pSG5(ts)}$), ori^{ColE1}, *sSpcas9*, synthetic guide RNA cassette	Cobb, Ryan E., et al. [70]
pCZ2	pSET152 Ω (lacZ::P*rpsL-dcas9* synthetic guide RNA cassette)	This study

Table S4. Oligonucleotides

Oligonucleotide	Sequence	Description
CZ107	CGCAAGTTGTACAGGCTGGGTCCG	*redD* sg2, *S. coelicolor*
CZ108	AACACGGACCCAGCCTGTACAACT	
CZ131	CGCAGTCCGGAGCCAGCCAAAGAT	*redD* sg2, *S. coelicolor*
CZ132	AACAATCTTTGGCTGGCTCCGGAC	
CZ133	CGCAAGGAGGCTCGAAGGCCGATA	*act*I-ORF1 sg1, *S. coelicolor*
CZ134	AACATATCGGCCTTCGAGCCTCCT	
CZ109	CGCAGTCGCCTTCGAGGATTTAAG	*act*II-ORF4 sg1, *S. coelicolor*
CZ110	AACACTTAAATCCTCGAAGGCGAC	
CZ101	CGCACGACCTCGGCAAAGGACGCG	*lnyI* sg1, *S*. Mg1
CZ102	AACACGCGTCCTTTGCCGAGGTCG	
CZ103	CGCATGCGGACGGCTGTAGTCGAG	*sfhA* sg1, *S*. Mg1
CZ104	AACACTCGACTACAGCCGTCCGCA	
CZ129	CGCAATTCCGAATGTCTTTGTCAT	*lnyHA*-sg1 gRNA, *S*. Mg1
CZ130	AACAATGACAAAGACATTCGGAAT	
CZ127	CGCAGGCTCCACAGAAACCCCTAA	*lnyHA*-sg2 gRNA, *S*. Mg1
CZ126	AACATTAGGGGTTTCTGTGGAGCC	
CZ194	CGCACAGTCAAACCAAATAGGGAT	*desA*-sg1 gRNA, *S*. Mg1
CZ195	AACAATCCCTATTTGGTTTGACTG	
CZ196	CGCAGAGCGCATCAACTTTCCTCC	*desA*-sg2 gRNA, *S*. Mg1
CZ197	AACAGGAGGAAAGTTGATGCGCTC	
CZ232	CGCACACTCTAACTTGAGCACGTT	Cluster 28 ORF-A sg1 gRNA, *S.* Mg1
CZ233	AACAAACGTGCTCAAGTTAGAGTG	
CZ234	CGCAGCGAGAAAGAAATGCCTGAT	Cluster 28 ORF-A sg2 gRNA, S. Mg1
CZ235	AACAATCAGGCATTTCTTTCTCGC	
CZ045	ACGCCGCGGTGACACCCGCGCAGG	Cluster 3 deletion gRNA, *S.* Mg1
CZ046	AAACCCTGCGCGGGTGTCACCGCG	
CZ040	ACGCAGACCACCGACGTACGGGTG	Cluster 9 deletion gRNA, *S.* Mg1
CZ039	AAACCACCCGTACGTCGGTGGTCT	
CZ061	ACGCTTTCGAGGTCGAAATCAGAG	Cluster 14 deletion gRNA, *S*. Mg1
CZ062	AAACCTCTGATTTCGACCTCGAAA	

Table S4. Oligonucleotides Continued

Oligonucleotide	Sequence	Description
CZ055	ACGCCGCGATGCTGGGGATCTGGG	Cluster 28 deletion gRNA, *S.* Mg1
CZ056	AAACCCCAGATCCCCAGCATCGCG	
CZ030	ACGCCGACCTCGGCAAAGGACGCG	*lnyI* deletion gRNA, *S.* Mg1
CZ031	AAACCGCGTCCTTTGCCGAGGTCG	
CZ032	TTGCCGCCGGGCGTTTTTTATCT AGAGACGTCTTCATCTCGCTGAC	*lnyI* deletion left HRD
CZ033	TTCGCTCCGTCAGGCCGTCACAT GATCCGTGAAATACCTTTGC	
CZ034	GCAAAGGTATTTCACGGATCAT GTGACGGCCTGACGGAGCGAA	*lnyI* deletion right HRD
CZ035	CCTTTTTACGGTTCCTGGCCTCT AGACCACCCTGCTCTTCGACCACC	
CZ041	TTGCCGCCGGGCGTTTTTTATCT AGACGGAGGTCCGAAGACTGCTG	Cluster 9 frameshift deletion left HRD
CZ042	CGAAGACCACCGACGTACGC CCGGTCCTTGCCGTATC	
CZ043	GATACGGCAAGGACCGGGCGT ACGTCGGTGGTCTTCG	Cluster 9 frameshift deletion right HRD
CZ044	CCTTTTTACGGTTCCTGGCCTCT AGAGGGAACGCCGTGAACAACTC	
CZ047	TTGCCGCCGGGCGTTTTTTATCT AGATCCCCTACCTGGAGCACTA	Cluster 3 frameshift deletion left HRD
CZ048	GATGCCGAGGTAGCCCTCCCGT AGTCGCTGTACATCAGC	
Iso-sgRNA-1	TCGGTCGCATGGCCGCCGGG	*crtY* sgRNA, *S.* Mg1
Iso-sgRNA-2	GGGCGCTTTACTGCATCCAC	
RMSq21	CTGCCTTCGTACCCGTCATG	*lnyHA-forward*
RMSq22	TGCGACCAGTTCACCTTCAG	*lnyHA-reverse*
RMSq23	CCTCGTCGACATCTACTCGC	*lnyHI-forward*
RMSq24	AGTCGAAGAACTGCTCCTGC	*lnyHI-reverse*
lvdAqF	GACAGCACCGTCTCCTTC	*lvdA-forward*
lvdAqR	GAGTACGGCGAGCAGTT	*lvdA-reverse*

Table S4. Oligonucleotides Continued

CZ049	GCTGATGTACAGCGACTACGGG AGGGCTACCTCGGCATC	Cluster 3 frameshift deletion right HRD
CZ050	CCTTTTTACGGTTCCTGGCCTCT AGACTCGGCAGGAGTGGGTCGA	
CZ057	TTGCCGCCGGGCGTTTTTATCT AGACGCCTACCTCACCCACTCCCG	Cluster 28 frameshift deletion left HRD
CZ058	GTTCGGCCGGGTCGATCAGGTT CCCCGGTGCGTTCGCACA	
CZ059	TGTGCGAACGCACCGGGGAACC TGATCGACCCGGCCGAAC	Cluster 28 frameshift deletion right HRD
CZ060	CCTTTTTACGGTTCCTGGCCTCT AGAGCCGTTTGCGGTCGACTTTCC	
CZ063	TTGCCGCCGGGCGTTTTTATCT AGACGTACAGCGCAGAAACTGAG	Cluster 14 frameshift deletion left HRD
CZ064	ATCACAAAAGGCCGAACAACCG GGAATCTCGTTGTTGTTC	
CZ065	AACAACAACGAGATTCCCGGTT GTTCGGCCTTTTGTGAT	Cluster 14 frameshift deletion right HRD
CZ066	CCTTTTTACGGTTCCTGGCCTCT AGAGAAGGTGAGGGTCTGGATGA	
CZ067	GACGGCGAGCTGACGTTCG	Cluster 28 deletion sequencing
CZ068	CGGCCAGGGCGTACTTCTC	
CZ069	GCTGCTGGCCCTGGAGATCTC	Cluster 9 deletion sequencing
CZ070	CAGCGCCCGCAGTCCGTAC	
CZ071	GGACTCGGGTGAGAGAGTCA	Cluster 14 deletion sequencing
CZ072	ATGAGCCATTCCACCTCAAG	
CZ119	GTGGTCACCCACTGTTTCGT	*lnyI* amplicon qRT-PCR primers
CZ120	GTACGGACAGCATGTGGAAG	
Q21	CTGCCTTCGTACCCGTCATG	*lnyHA* amplicon qRT-PCR primers
Q22	TGCGACCAGTTCACCTTCAG	
CZ028	CTTCGGTCGGACGTGCGTCTAC	Sequencing primers for pCRIPSPomyces-2 assembly
CZ029	CGAGGGAGCTTCCAGGGGGAAA	

Table S5. Quantitative RT-PCR of *lnyHA* and *lnyHI* transcripts.*

lnyHA	**‡ Mean DDCq (*lnyHA*)**	***lnyHA* fold change(2^{-DDCq})**
sg2	-0.57±0.97	1.48±1.5
sg1+2	3.53±0.81	0.09±0.05
lnyHI	**‡ Mean DDCq (*lnyHI*)**	***lnyHI* fold change(2^{-DDCq})**
sg2	-1.10±0.88	2.14±0.9
sg1+2	1.43±0.43	0.37±0.5

* *hrdB was used as the reference gene for rt-PCR.*

‡ *ΔΔCq values and fold changes are relative to the vector control strain. rt-PCR was carried out with 3 replicates for each gene.* $DCq_{target} = Cq_{target} - Cq_{hrdB}$ *and Mean* DCq_{target} *was determined. Mean* $DDCq_{target}$ = *Mean* DCq_{target}-*Mean* DCq_{pVect}. *Fold change was calculated as* $2^{(-DDCqtarget)}$.

Table S6. Quantitative RT-PCR of *lvdA* transcripts.*

lvdA	**‡ Mean DDCq (*lvd*A)**	***lvdA* fold change(2^{-DDCq})**
sg2+3	-1.27±0.68	2.41±0.9
sg1+2	6.28 ± 0.41	0.013±0.004

* *hrdB was used as the reference gene for rt-PCR.*

‡ *ΔΔCq values and fold changes are relative to the vector control strain. rt-PCR was carried out with 3 replicates for each gene.* $DCq_{target} = Cq_{target} - Cq_{hrdB}$ *and Mean* DCq_{target} *was determined. Mean* $DDCq_{target}$ = *Mean* DCq_{target}-*Mean* DCq_{pVect}. *Fold change was calculated as* $2^{(-DDCqtarget)}$.

Supplemental Methods:

Quantitative real-time PCR (qRT-PCR)

Total RNA was extracted from Streptomyces cultures by bead-beating using 300 μm acid-washed glass beads, followed by extraction with TRI Reagent RT (MRC) according to the manufacturer's protocol. Total RNA was treated with RNase-Free DNase I kit (Thermo fisher). The isolated RNA was quantified with Qubit fluorometer using Qubit broad range RNA assay kit (Thermo Fisher). 500 ng of the total RNA was converted to cDNA using High Capacity RNA-to-cDNA kit (Thermo Fisher). Quantitative PCR was carried out using PowerTrack SYBR green Master Mix (Thermo Fisher) and CFX96 Touch real-time PCR thermocycler (Bio-Rad). The thermal cycling parameters used were as follows: denaturation at 95 ˚C for 30 s; 40 cycles of denaturation at 95 ˚C for 15 s, annealing at 56 ˚C for 30 s, and extension at 72 ˚C for 30 s; followed by a final melting curve from 60-95 ˚C for 6 min.

We amplified *lnyHA, lnyHI*, the first and the last open reading frames in the linearmycin BGC and *lvdA*, the first open reading frame in the lavendomycin BGC using the primers mentioned in Table S4.

References:

(1) Schwyn, B.; Neilands, J. B. Universal Chemical Assay for the Detection and Determination of Siderophores. *Analytical Biochemistry* **1987**, *160* (1), 47–56. https://doi.org/10.1016/0003-2697(87)90612-9.

(2) Komori, T.; Ezaki, M.; Kino, E.; Kohsaka, M.; Aoki, H.; Imanaka, H. Lavendomycin, a New Antibiotic. I. Taxonomy, Isolation and Characterization. *The Journal of antibiotics* **1985**, *38* (6), 691–698. https://doi.org/10.7164/antibiotics.38.691.

(3) Cediel-Becerra, J. D. D.; Cumsille, A.; Guerra, S.; Ding, Y.; Crécy-Lagard, V. de; Chevrette, M. G. Targeted Genome Mining with GATOR-GC Maps the Evolutionary Landscape of Biosynthetic Diversity. *bioRxiv* **2025**, 2025.02.24.639861. https://doi.org/10.1101/2025.02.24.639861.
